# Surface-modified CMOS biosensors

**DOI:** 10.3389/fbioe.2024.1441430

**Published:** 2024-11-06

**Authors:** Fahimeh Dehghandehnavi, Md. Sakibur Sajal, Marc Dandin

**Affiliations:** Electrical and Computer Engineering Department, Integrated Circuits and Bioengineering Laboratory, Carnegie Mellon University, Pittsburgh, PA, United States

**Keywords:** complementary metal-oxide-semiconductor (CMOS), biosensor, immobilization, post-CMOS process, transduction, lab-on-a-chip (LOC)

## Abstract

Biosensors translate biological events into electronic signals that quantify biological processes. They are increasingly used in *in vitro* diagnostics applications that leverage their ability to process small sample volumes. One recent trend has been to integrate biosensors with complementary metal-oxide-semiconductor (CMOS) chips to provide enhanced miniaturization, parallel sensing, and low power consumption at a low cost. CMOS-enabled biosensors are used in monitoring DNA hybridization, enzymatic reactions, and cell proliferation, to name a few applications. This paper explores the materials and processes used in emerging CMOS biosensors. We discuss subtractive and additive processes for creating electrodes for electrochemical sensing applications. We discuss functionalization techniques for creating bioelectronic interfaces that allow molecular events to be transduced into the electrical domain using a plurality of modalities that are readily provided by CMOS chips. Example modalities featured are optical sensing, electrochemical detection, electrical detection, magnetic sensing, and mechanical sensing.

## 1 Introduction

**FIGURE 1:**
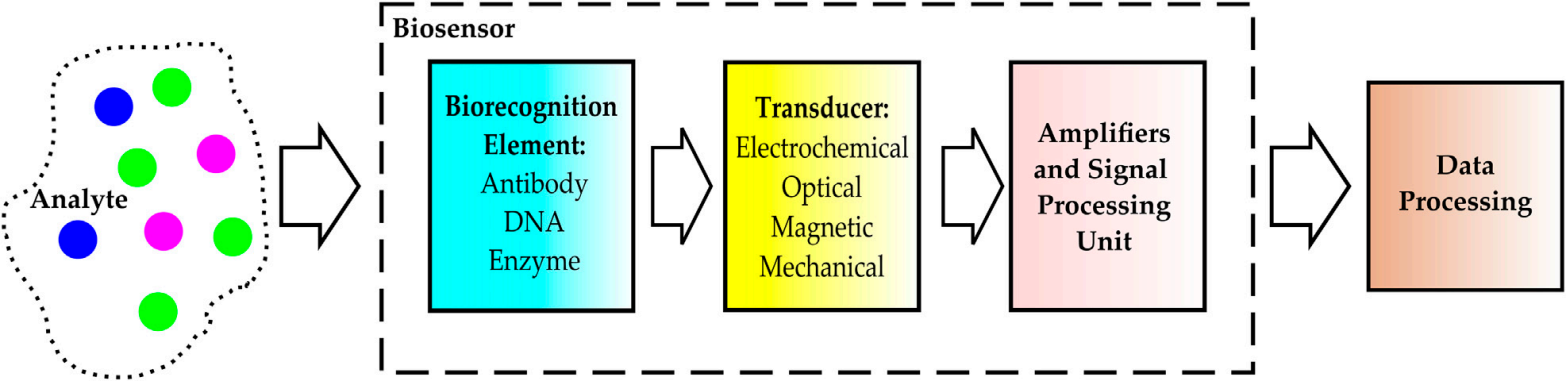
Schematic representation of biosensors’ working principle.

Biosensors are devices that detect chemical compounds from specific biochemical reactions or events mediated by enzymes, immunosystems, tissues, organelles, or whole cells. Biosensors translate these reactions into electrical, thermal, or optical signals (Dellweg et al., 1992). Three main components make a biosensor: one or multiple transducers, biorecognition elements (BREs), and the required signal processing systems. The transducer generates target-specific signals upon target-BRE interactions. These signals are amplified and processed and then sent to a computer for data analysis (Figure 1) (Grieshaber et al., 2008; Jang and Hassibi, 2009; Syu et al., 2018). Biosensors are widely used across multiple disciplines, from healthcare and medical diagnostics to environmental sciences.

One application of biosensors is in *in vitro* diagnostics (IVD) where they are used to verify the presence of biological target analytes (e.g., DNA, protein, bacteria, etc.). IVD offers the benefits of high precision alongside considerable convenience for patients, as it requires only small-scale biological samples. However, IVD tools are bulky and expensive and require trained personnel to operate. These drawbacks hinder accessibility and lengthen turnaround times. These challenges can be more problematic in developing countries and in the case of contagious diseases like the recent COVID–19 global pandemic.

In response to such cases, the concepts of decentralized healthcare and point-of-care (POC) medicine have gained great attention, resulting in the need for smaller, more cost-effective biosensors. For biosensors to be effective in POC settings, they must maintain high sensitivity and selectivity while achieving low power consumption, a small footprint, and minor sample usage (Lei et al., 2016; Thriveni and Ghosh, 2022). Complementary metal-oxide-semiconductor (CMOS) technology meets these criteria.

CMOS technology—used in building integrated circuits (ICs) and chips—allows the integration of the transducer, which may be a photodiode (PD), an ion-sensitive field-effect transistor (ISFET), a metal-oxide-semiconductor field-effect transistor (MOSFET), or a nanowire field-effect transistor (NWFET), etc., and signal processing units on a single chip. With the signal amplification and processing circuitry on the same chip as the transducer, generated signals (e.g., current and voltage) can be handled directly. In addition to making the sensor low-cost and portable, this integration reduces external noise and improves the signal-to-noise ratio (SNR) and the limit of detection (LOD) (Lei et al., 2016; Syu et al., 2018). However, to enhance sensitivity and selectivity, CMOS chips integrated into biosensors can benefit from two types of surface modification: post-CMOS micro- and nano-fabrication processes, and wet biochemical procedures.

These surface modifications are particularly important because they facilitate molecular detection at lower concentrations, enabling earlier diagnosis. Moreover, enhancing the LOD in CMOS biosensors is a step toward molecular detection using typically unused biofluids like sweat. Many biomarkers and biomolecules in human blood, such as inflammatory markers like Tumor Necrosis Factor-alpha (TNF- α ) and Interleukin-6 (IL-6), and biomolecules like glucose and cortisol, are present in sweat as well, and their concentration in sweat is an indicator of their concentration in blood. However, the concentration of these biomarkers in sweat is much lower than their concentration in blood, which makes detection more challenging. By improving CMOS biosensors’ LOD, biomolecules can be detected through sweat, enabling non-invasive and continuous monitoring (Zafar et al., 2022; Hirten et al., 2024; Kothari et al., 2022).

Below, we provide a brief overview of how post-CMOS processes and wet biochemical procedures enhance CMOS biosensors’ performance. Circuit components and metal interconnects of ICs fabricated with CMOS technology are buried in an oxide layer ( ${SiO}_{2}$ ) for insulation and topped with a passivation layer ( ${SiO}_{2}$ /S $i_{3}N_{4}$ ) for protection. Employing post-CMOS processes to selectively remove or thin the oxide and passivation layers, deposit a biocompatible layer on the chip’s surface, or fabricate on-chip electrodes, expands the versatility of CMOS biosensors (Huang et al., 2021; Jang et al., 2009). For example, (Al-Rawhani et al., 2020) performed plasma-enhanced chemical vapor deposition (PECVD), electron beam lithography, and electron beam evaporation on a CMOS chip to build a rapid biomarker detector; (Lai et al., 2012) used RF sputtering to deposit ${RuO}_{2}$ on a polyethylene terephthalate (PET) substrate; (Lin C. H. et al., 2022) thinned the top passivation and inter-metal dielectric layers of their CMOS capacitive sensor by dry etching to improve sensitivity; (Doi et al., 2022; Kuo et al., 2020; Hsu et al., 2018; Wang et al., 2022b) sputtered noble metals on a CMOS chip’s top metal layer; (Hu et al., 2021) chemically etched away the aluminum (Al) top metal layer to expose the electrodes’ underlying titanium nitride (TiN) and used the exposed TiN for pH and impedance sensing.

**FIGURE 2:**
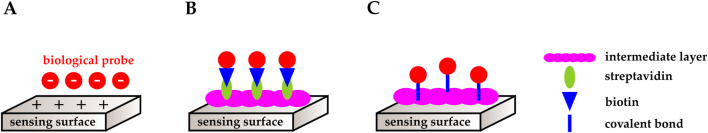
Schematic representation of functionalization mechanisms. **(A)** Physical adsorption. **(B)** Streptavidin-biotin complex. **(C)** Covalent immobilization.

Furthermore, to improve sensitivity and selectivity, the surface of CMOS chips may need to be functionalized with proper biological probes. For example, in affinity-based biosensing, biosensing elements such as antibodies, receptor proteins, or DNA are immobilized on the transducer’s solid surface. Target analytes interact with the capture layer and bind to the immobilized probes, and the transducer translates these biological interactions into quantifiable signals (e.g., electrical, optical, mechanical, and magnetic) (Jang and Hassibi, 2009; Lei et al., 2016; Devadhasan et al., 2015; Rogers, 2000). For example, Manickam et al. (2017) deposited (3–glycidoxypropyl) trimethoxysilane on a CMOS photodiode via chemical vapor deposition (CVD) to further immobilize synthetic DNA probes on the chip’s surface. Various techniques have been studied and employed for probe immobilization, including physical adsorption (Figure 2A), streptavidin-biotin interaction (Figure 2B), and covalent immobilization (Figure 2C). We briefly describe some of these methods below.

Physical adsorption is a non-covalent functionalization method that relies on the ionic interactions between biological probes and the sensing surface (Figure 2A). The main advantage of this method is simplicity, as it does not require linker molecules or modifications to the capture probe and the target analyte (Nimse et al., 2014). Physical adsorption is a good method for DNA probe immobilization as DNAs are negatively charged. One example is in (Moser et al., 2018).

The use of streptavidin-biotin complexes is another non-covalent approach to functionalization. In this method, the sensing surface is functionalized with streptavidin using an intermediate layer, and the capture probe is labeled with biotin (Figure 2B). The streptavidin-biotin complex has one of the strongest non-covalent bonds, while biotin is a stable label with minimal interference with the labeled molecule’s functionality. This is a well-established technique for detecting proteins and DNA (Nimse et al., 2014; Smith et al., 2005), and an example can be found in (Crucifix et al., 2004).

Another method of functionalization employs covalent bonds. Covalent immobilization provides stronger binding and better stability compared to physical adsorption and streptavidin-biotin-based immobilization. In this method, the adherence of the biosensing element to the sensing surface is achieved through covalent bonds. Similarly to the streptavidin-biotin technique, covalent immobilization involves an intermediate layer between the sensing surface and the capture probe (Figure 2C). In biosensors with gold electrodes, for example, a common immobilization technique is modifying the electrode or the capture probes with thiol groups (RSH), which are present in cysteine amino acids, taking advantage of their strong affinity toward noble metals (Nimse et al., 2014). This technique was used in (Kuo et al., 2021; Lee et al., 2012). As another example, silanization is the preferred immobilization method in oxide sensing membranes (Pinitwong and Pijitrojana, 2015). In silanization, 3–aminopropyltriethoxysilane (APTES) and glutaraldehyde (GA) are commonly used in the crosslinking process. APTES is a self-assembled monolayer that binds to the transducer’s surface. Its amine group provides a platform for the bifunctional reagent, GA, to form covalent bonds to the capture probe (Syu et al., 2018; Nimse et al., 2014). Examples of biosensors that benefit from the APTES-GA immobilization technique are reported in (Lin C. H. et al., 2022; Zhou et al., 2021; Huang et al., 2013b).

Although advances in post-CMOS processes and wet biochemical procedures have made it possible to modify CMOS chips’ surfaces, there are challenges in this emerging field that must be addressed before moving from a research phase to full commercialization. For example, post-CMOS processes need large-scale facilities and are expensive to perform. Additionally, the biochemical procedures employed to immobilize biological probes on the CMOS biosensors include exposing the chip to harsh conditions such as low pH and elevated temperature, which may reduce chip reliability.

This paper provides a critical and comprehensive review of recently developed CMOS biosensors with surface modification and functionalization. We cover a variety of sensors and functionalization techniques. We also discuss the advantages and disadvantages of each method, the remaining challenges and shortcomings, and future trends in the field. A performance summary of 60+ surface-modified CMOS biosensors developed since the year 2003 is presented in Supplementary Tables 1–3 in the Information section of the paper.

## 2 CMOS electrochemical biosensing

Electrochemical sensing may be defined as the transformation of analyte-electrode interactions into a comprehensible signal (Hulanicki et al., 1991). Since the introduction of the first enzyme-electrode biochemical-based biosensor by (Clark and Lyons, 1962), electrochemical sensing has gained more attention in biosensing applications. Today, electrochemical sensing is favored because of its fast response, low cost, lack of complexity, and potential for miniaturization using nanotechnology. Additionally, electrochemical biosensing allows label-free detection, which adds to its adaptability (Li et al., 2017).

**FIGURE 3:**
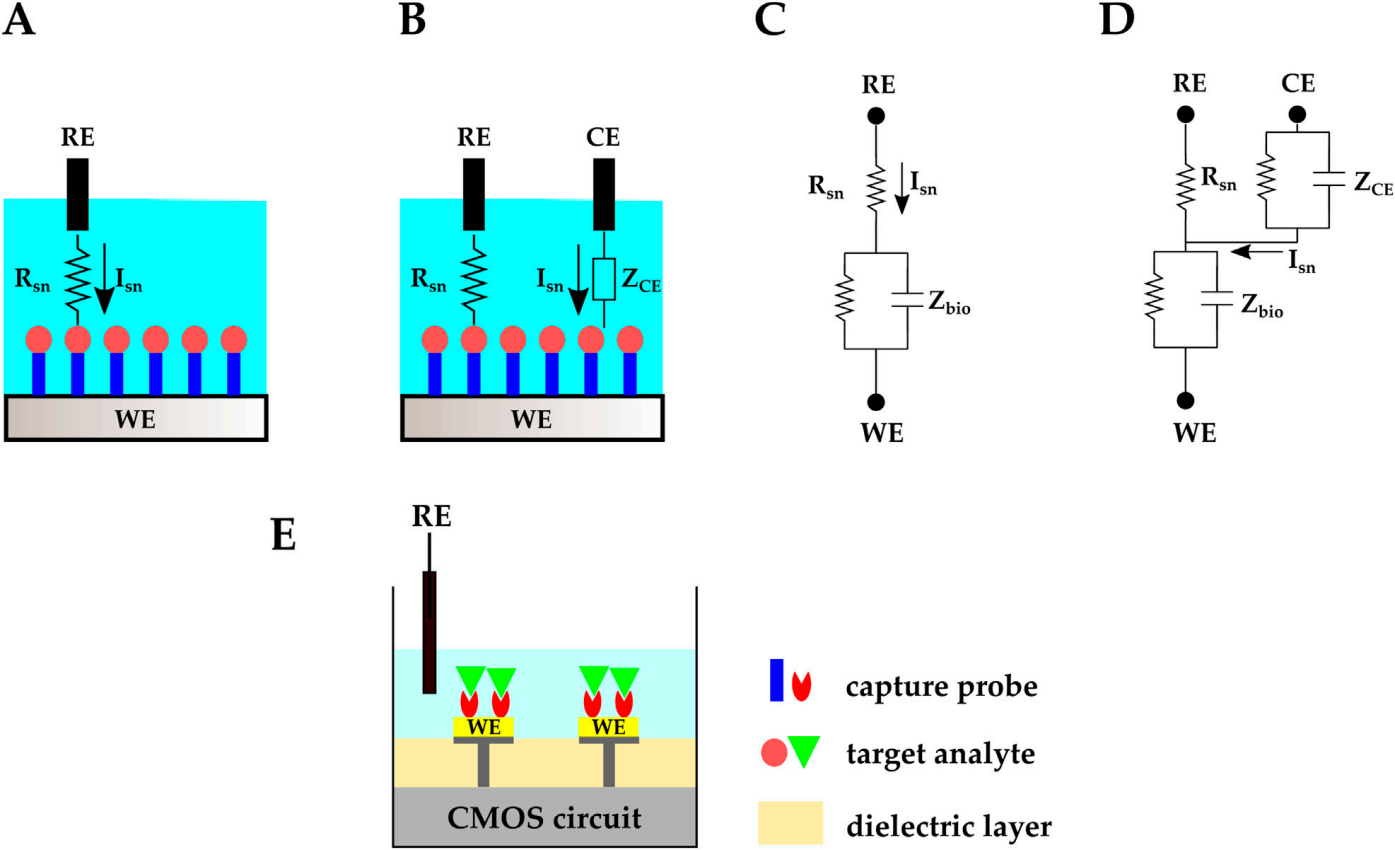
Schematic representation and circuit equivalent of **(A, C)** two-electrode and **(B, D)** three-electrode electrochemical biosensing. **(E)** CMOS electrochemical biosensor. $R_{sn}$ is the solution equivalent resistance between the RE and the BRE. $Z_{CE}$ is the equivalent impedance between the CE and the RE. $I_{sn}$ is the current going through the solution. $Z_{\mathrm{bio}}$ represents the BRE equivalent impedance.

In electrochemical biosensors, the analyte resides in a gas or liquid environment that supports the analyte’s biological activity and its transport to electrodes or sensing membranes. The electrode or sensing membrane is sensitive to the target analyte and transduces the electrochemical reactions into electrical signals. This electrode is referred to as the working electrode (WE). In two-electrode electrochemical biosensors, a reference electrode (RE) provides a known stable DC voltage, and the WE potential is set/read with respect to this RE (Figures 3A, C). Another electrochemical sensing platform includes a third electrode, called the counter electrode (CE). The CE is used in Faradaic electrochemical biosensors–where a significant current is created due to the electrochemical reactions–and provides proper biasing to compensate for the voltage drop across the electrolyte’s equivalent resistance (Figures 3B, D) (Li et al., 2017).

In conventional electrochemical biosensors, input and output electrical signals (e.g., voltage and current) are handled with a benchtop potentiostat. CMOS technology has made it possible to integrate the electrochemical biosensor’s electrodes and the readout circuit on one chip, eliminating the need for an external potentiostat (Figure 3E). Examples of CMOS potentiostats are in (Zhang et al., 2005; Honarvar Nazari and Genov, 2009; Li et al., 2021). Moreover, with CMOS technology, microelectrode array (MEA) structures can be fabricated to serve as electrodes. These MEAs provide high spatial resolution and enable high-throughput platforms with parallel sensing capabilities.

One of the main challenges in developing CMOS electrochemical biosensors is the required post-CMOS processes. Aluminum used in CMOS processes is not suitable for biosensing, and polarizable metals such as Pt and Au are not compatible with CMOS technology. Additive and subtractive procedures have been performed to create biocompatible electrodes suitable for electrochemical applications (Huang et al., 2021). It should be mentioned that fabricating REs with CMOS technology is still challenging and external Ag/AgCl REs are commonly employed in CMOS electrochemical biosensors (Zhang et al., 2019; Chang and Lu, 2020; Saengdee et al., 2021; Doi et al., 2022; Lin C. H. et al., 2022).

The transducer electrode in an electrochemical biosensor translates the target electrochemical reaction into electrical signals, which can be in the form of current or voltage. Based on the output signal type, electrochemical sensors are often classified as amperometric/voltammetric, potentiometric, and impedimetric (Lin C.-Y. et al., 2022). There is also another major category based on field-effect transistors (FETs). In the following subsections, we will discuss surface-modified CMOS electrochemical biosensors based on these classifications.

### 2.1 Amperometric/voltammetric biosensing

**FIGURE 4:**
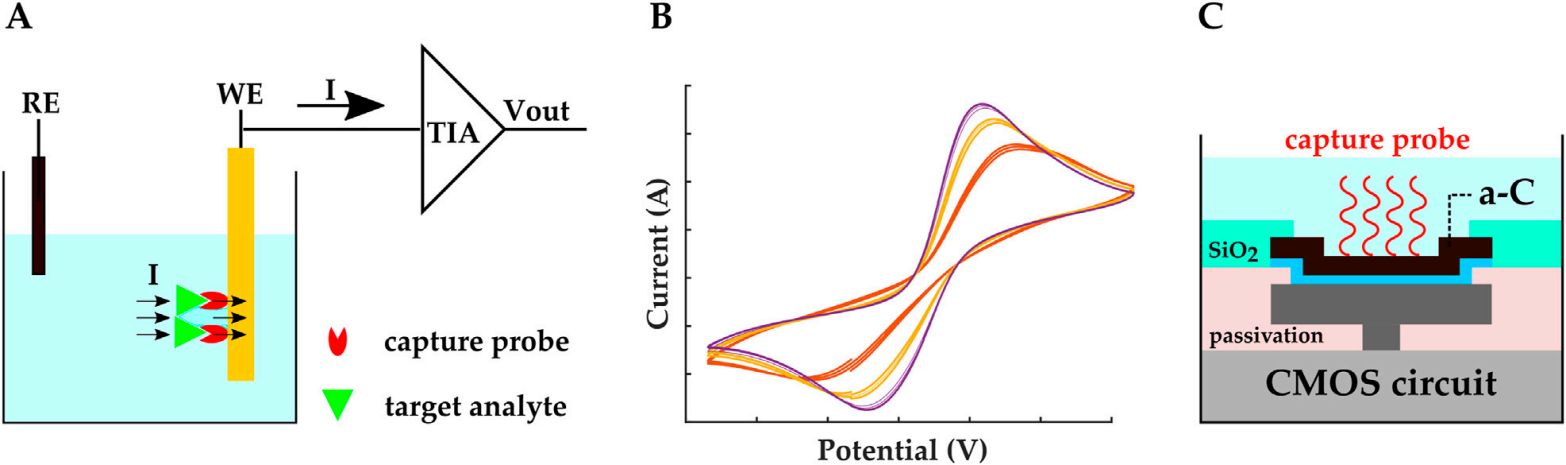
Schematic representation of **(A)** amperometric/voltammetric electrochemical biosensor. The TIA is a transimpedance amplifier. **(B)** An example of cyclic voltammetry I-V plot. **(C)** Amperometric CMOS biosensor developed by (Manickam et al., 2019a; b). Electrodes were realized by covering the exposed top metal layer with a-C. A 10 nm Ti layer was used as the adhesion layer. MB-labeled stem-loop DNA hairpin probes were covalently immobilized on the a-C electrodes.

Amperometry is a Faradaic electrochemical sensing method that requires redox molecules. In this method, the WE is set at a constant voltage with respect to the RE. This controlled potential initiates oxidation or reduction reactions, resulting in an electric current (Figure 4A) that is associated with the analyte’s concentration through the Cottrell equation (Equation 1). In Equation 1, *i* is the current (Ampere), *n* is the number of electrons, *F* is the Faraday constant, *A* is the electrode’s area ( ${cm}^{2}$ ), $c_{j}^{0}$ is the reducible analyte’s initial concentration (mol/ ${cm}^{3}$ ), $D_{j}$ is the species diffusion coefficient ( ${cm}^{2}$ /s), and *t* is time (sec) (Hulanicki et al., 1991; Li et al., 2017; Baranwal et al., 2022).

$$i=\frac{nFAc_{j}^{0}\sqrt{D_{j}}}{\sqrt{\pi t}}$$ (1)

In cyclic voltammetry (CV), the applied voltage between the WE and the RE is ramped, and plots of the resulting bidirectional current vs. the applied ramped voltage are retrieved, with oxidation and reduction peaks appearing in these I-V plots (Figure 4B). Different species lead to different peaks; hence, peaks can be studied to distinguish multiple analytes (Huang et al., 2021). Similar to amperometry, CV is a Faradaic sensing mechanism, and it requires redox molecules to be present in the solution.

Amperometry and CV are commonly used to study the exocytotic release of neurotransmitter biomolecules such as acetylcholine, dopamine (DA), glutamate, serotonin, etc. Conventional electrochemical sensors use carbon fiber microelectrodes for this application (Mosharov and Sulzer, 2005); however, carbon fiber microelectrodes allow measurements from one cell at a time, which makes the measuring process tedious and lengthy (Huang et al., 2021). In contrast, CMOS technology provides a platform for integrating thousands of MEAs on a single chip, enabling parallel sensing. For example, a three-electrode fast-scan CV (FSCV) CMOS-graphene dopamine sensor was introduced in (Nasri et al., 2017), in which carbon microfiber wires were replaced with ultrathin planar multilayer graphene sheets. To make the graphene electrodes, an epitaxial graphene film was transferred onto the CMOS chip, and after multiple lithographic and metal deposition steps, graphene microelectrodes were realized and connected to the readout circuit. *In vitro* measurements for a 2 μM DA release were performed to validate the sensor’s performance and sensitivities of 8–14 nA/μM were achieved for 200–400 V/s ramp rates.

Monitoring DNA hybridization is another biosensing application of amperometry and CV. For example (Manickam et al., 2019a; b), developed a CMOS three-electrode voltammetric electrochemical biosensor for molecular diagnostics (MDx) applications. This biochip included an array of 32×32 pixels. Each pixel contained a transducer and its ΣΔ front-end circuitry. The WE, RE, and CE were realized by exposing the electrodes, made of the top metal layer, and covering them with amorphous carbon (a-C). The a-C electrodes were further functionalized with DNA hairpin probes labeled with methylene blue (MB) (Figure 4C). Upon adding the complementary DNA sequences, the probes captured the target sequence and the hairpins opened consequently, pushing the MB away from the electrode’s surface, resulting in a decrease in the sensing current. The lower LOD and sensitivity for this biochip was reported to be 70 nM and 4 pA /μ M, respectively.

### 2.2 Potentiometric biosensing

**FIGURE 5:**
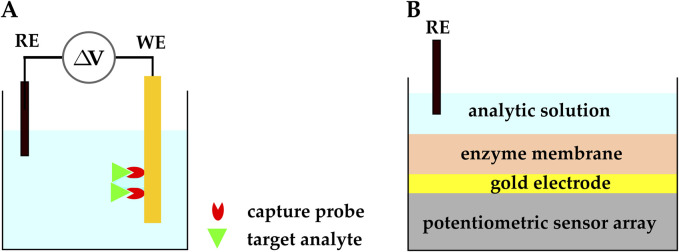
**(A)** Schematic representation of a potentiometric electrochemical biosensor. Δ V is an open-circuit voltage between RE and WE.**(B)** An ISE-based potentiometric CMOS biosensor developed by (Doi et al., 2022).

In the potentiometric electrochemical sensing approach, the WE potential is measured against the RE when no significant current flows between the two electrodes (Figure 5A). The measured potential at the WE relates to the analyte concentration *via* the Nernst equation (Equation 2). In Equation 2, $E_{\mathrm{cell}}$ is the measured cell potential when no current exists (V), $E_{\mathrm{cell}}^{0}$ is the standard cell potential (V), *R* is the universal gas constant (J/(K.mol)), *T* is the temperature (K), *n* is the number of transferred electrons, *F* is the Faraday constant (C/mol), and *Q* is the anode-to-cathode ion concentration (Stradiotto et al., 2003; Grieshaber et al., 2008; Walker et al., 2021).

$$E_{\mathrm{cell}}=E_{\mathrm{cell}}^{0}-\frac{RT}{nF}lnQ$$ (2)

Potentiometric electrochemical sensors may have two types of sensing electrodes: ion-selective electrodes (ISEs) and field-effect transistors (FETs). ISEs are the typical choice of electrode for potentiometric sensors, and they have been used since the 1990s. More recently, FETs have been employed as the sensing element in potentiometric sensors because of their ion sensitivity and potential for miniaturization. While CMOS FET-based biosensors have contributed a lot to the field (Ding and Qin, 2020; Walker et al., 2021; Wu et al., 2023), they are beyond the scope of this section; hence, we discuss FET-based biosensors in more detail in Section 2.4, and we focus on ISE-based biosensors in this section.

As mentioned above, potentiometric electrochemical sensors typically use ISEs and ion-selective membranes as their sensing platform. ISE-based potentiometric sensors have traditionally been used as pH sensors; however, with advances in fabrication and functionalization methods, they have found applications in biosensing and POC medicine. Recently, CMOS-based ISEs have been functionalized with biosensing elements and used to detect biomolecules such as enzymes, nucleic acids, and proteins (Ding and Qin, 2020; Walker et al., 2021; Wu et al., 2023). For example, a CMOS potentiometric biosensor with functionalized ISEs was developed for the monitoring of extracellular Adenosine $5^{\prime }$ –triphosphate (ATP) through hydrogen ion imaging (Doi et al., 2022).

ATP plays a major role in the central nervous system as it contributes to synaptic transmission regulation and is correlated with neurodegenerative diseases and psychiatric disorders. In the presence of ATP, hydrogen peroxide ( $H_{2}O_{2}$ ) is generated through enzymatic reactions from glycerol-kinase (GK) to L– α –glycerophosphate oxidase (LGOx) to horseradish peroxidase (HRP). As a result, ATP can be detected through changes in $H_{2}O_{2}$ concentration (Cao et al., 2013; Fellin et al., 2006; Gourine et al., 2005; Koizumi et al., 2003).

The sensor implemented in (Doi et al., 2022) was realized by depositing gold electrodes on a CMOS charge-transfer-based potentiometric sensor array, which included 128×128 pixels with 37.3 μ m spatial resolution. The circuitry of the sensor is discussed in (Futagawa et al., 2013). GK, LGOx, and HRP were encapsulated in a polymer film immobilized on the gold electrodes using a mixed-layered technique based on physical adsorption. The analytic solution was placed on top of this polymer film and an external Ag/AgCl electrode was immersed in the solution to function as the RE (Figure 5B). The ISE-based potentiometric biosensor provided information on the spatial and temporal dynamics of extracellular ATP through sensing $H_{2}O_{2}$ and successfully detected ATP with LOD of 2.8 μ M and sensitivity of 77 ± 3.8 mV/dec for concentrations below 10 mM.

### 2.3 Impedimetric biosensing

**FIGURE 6:**
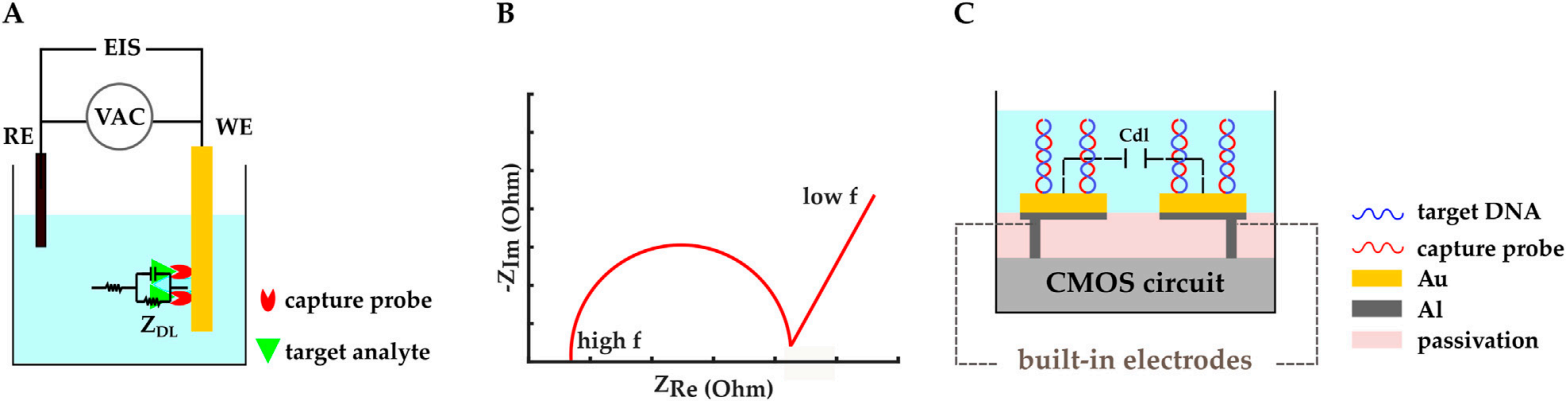
**(A)** Schematic representation of an impedimetric electrochemical biosensor. ZDL and EIS are double-layer impedance and electrochemical impedance spectroscopy, respectively. **(B)** An example Nyquist plot. **(C)** A CMOS capacitive biosensor designed by (Kuo et al., 2020). Electrodes were initially made with the top metal layer and they were covered by Au in post-processing. Thiolated microRNA–195 probes were immobilized on the gold IDE. Cdl represents the double layer capacitance.

Electrochemical impedance spectroscopy (EIS) is another widespread biosensing method in which the electrode-electrolyte surface is perturbed by an AC voltage or current with small enough amplitudes that the system stays in the linear region. By varying the stimulation signal frequency and studying the resultant current or voltage, the impedance of the electrochemical system can be retrieved as a function of frequency (Figure 6A) (Lei et al., 2016; Li et al., 2017; Dorledo de Faria et al., 2019; Forouhi and Ghafar-Zadeh, 2020; Lin C. H. et al., 2022). Nyquist plots are used to interpret the complex impedance of the electrochemical cell by representing the negative of the imaginary vs real parts of the impedance (Figure 6B).

EIS can be performed in Faradaic and non-Faradaic modes. Faradaic EIS relies on redox probes to study charge transfer resistance upon biological interactions, whereas non-Faradaic EIS does not need additional redox probes, which makes it better suited for POC applications (Lei et al., 2016; Li et al., 2017; Dorledo de Faria et al., 2019; Forouhi and Ghafar-Zadeh, 2020; Lin C. H. et al., 2022). For example, capacitive sensing is a non-Faradaic EIS method that is widely used in POC biosensors.

Capacitive sensing is commonly employed in affinity-based biosensing, where the BRE (e.g., DNA, aptamer, antibody, etc.) is immobilized on the surface of the capacitor electrodes, so the target analyte (e.g., DNA, protein, antigen, etc.) binds to the BRE. The binding event modulates the electrical double layer (EDL) and the double-layer capacitance ( $C_{dl}$ ) at the electrode-electrolyte interface. Changes in the double-layer capacitance can be detected from the measured impedance through EIS. It should be noted that the double-layer capacitance is a valuable impedance component because it is only related to the charge carriers and their concentration in the electrode-electrolyte boundary layer (Lei et al., 2016; Dorledo de Faria et al., 2019; Lin C.-Y. et al., 2022). Below are examples of recently developed CMOS capacitive biosensors.

A CMOS capacitive biosensor with interdigitated electrodes (IDEs) was designed, fabricated, and tested by (Kuo et al., 2020) to detect the breast cancer biomarker, microRNA–195. Gold IDEs were fabricated on the CMOS readout chip in the form of two separate combs, and complementary thiolated DNA probes were immobilized on the gold IDEs (Figure 6C). It was observed that the shift in the output frequency and the IDEs’ capacitance value increased as the microRNA-195 concentration was increased. The reported LOD, sensitivity, and dynamic range of the optimal CMOS IDE design were 0.617 fM, 645 Hz/fM, and 10 fM – 10 pM, respectively.

Another example is in (Lin C. H. et al., 2022), where a capacitive CMOS biosensor was developed to detect the Hepatitis B virus (HBV). The IDEs of the biosensor were formed by the top aluminum layer buried in the inter-metal dielectric layer and under the passivation layer. To increase the sensitivity, the passivation and inter-metal dielectric layers were thinned in a dry etching post-CMOS process. The chip’s surface was then functionalized with HBV probe DNA via an improved silanization method where 3–aminopropyldimethylethoxysilane (APDMES) was used instead of APTES. Different from APTES, each APDMES molecule reacts with one silanol group alone and prevents self-crosslinking and aggregation across the monolayer. They successfully demonstrated detection of HBV DNA hybridization with 10.8 fF/log [DNA] sensitivity in the 1–100 fM range.

In another study, Tabrizi et al. proposed a CMOS capacitive biosensor for DNA nano-mass measurement (Tabrizi et al., 2021). In this biosensor, DNA storage wells were realized by selectively etching the oxide and passivation layers to reach the top metal aluminum layer. The Al layer turned into ${Al}_{2}O_{3}$ upon getting exposed, and no further surface modification was needed as ${Al}_{2}O_{3}$ is known to be compatible with DNA molecules. The biochip’s capacitance was measured before and after having DNA samples in the wells, and the sensor successfully measured the DNA nano-mass with 18.5 aF/ng sensitivity.

### 2.4 Field-effect transistor-based biosensing

**FIGURE 7:**
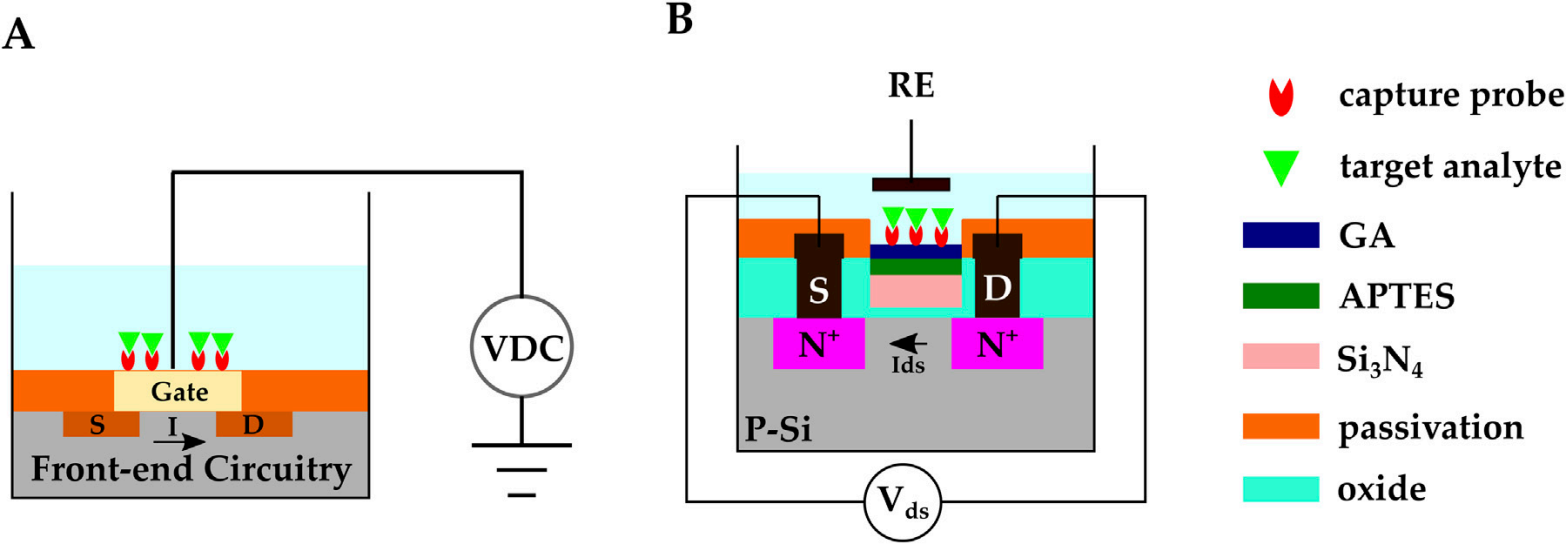
**(A)** Schematic representation of FET-based CMOS electrochemical biosensor **(B)** A CMOS ISFET-based biosensor developed by (Saengdee et al., 2021). Anti-HSA was covalently bonded on the ${Si}_{3}{Ni}_{4}$ passivation layer through the APTES-GA method to function as the sensing membrane. An Ag/AgCl electrode was used as the RE. The ${Si}_{3}{Ni}_{4}$ -ISFET current drain was kept constant and the gate potential was monitored.

Most bio-recognition events involve an electrostatic charge transfer, and subsequently, electric potential variations across the electrode-electrolyte system. Because FETs are sensitive to their gate potential voltage, they stand out for electrochemical biosensing applications. Moreover, FETs can be fabricated with CMOS technology (Figure 7A), offering mass production capability and low-cost manufacturing.

Biomolecules are detected in FET-based biosensors through two main mechanisms: 1) The FET channel surface is functionalized with the capture probes. Target species bind to the channel surface and alter the channel’s conductivity through the charge transfer process. The presence of biomolecules can be detected by studying the FET’s source-drain current ( $I_{D}$ ). 2) Target species are detected through changes in the FET’s dielectric capacitance and its threshold voltage ( $V_{th}$ ). In this method, the BRE is immobilized in a cavity adjacent to the gate dielectric layer. Upon binding of the biomolecules to the BRE, the gate dielectric constant changes, resulting in a change in gate oxide capacitance ( $C_{ox}$ ) and $V_{th}$ . The shift in $V_{th}$ can be observed in the $I_{D}$ plots (Ambhorkar et al., 2018).

It should be noted that standard planar gate FETs are in fact not sensitive enough to detect chemically or biologically charged species, and advances in post-CMOS processes have made it possible to fabricate FETs with special structures to achieve the required ultra-sensitivity. For example, the CMOS process was followed by a MEMS process in (Ushijima et al., 2021). These types of post-CMOS fabrication processes have allowed ISFETs (Bergveld, 1970) and nanowire FETs. These are discussed further below in Section 2.4.1, Section 2.4.2.

#### 2.4.1 Biosensing with ion-sensitive field-effect transistors

ISFET-based electrochemical sensors are one of the common types of potentiometric biosensors. The conventional FET with a MOSFET includes a gate, a source, a drain, and a body, and the metal gate is mainly employed as the WE and BRE immobilization platform; whereas in ISFETs, an oxide functions as the sensing membrane (e.g., ${SiO}_{2}$ , ${RuO}_{2}$ , etc.) (Forouhi and Ghafar-Zadeh, 2020; Syu et al., 2018). Changes in the ionic concentrations of the solution in contact with the oxide sensing membrane result in a change in ISFET’s threshold voltage and gate capacitance. These changes can be detected from alterations in the ISFET current (Al-Rawhani et al., 2020).

Lee et al. proposed a novel ISFET-based biosensor for DNA hybridization detection. They etched the passivation layer so that only a thin oxide layer remained covering the gate (Lee et al., 2021). The thin oxide on top of the ISFET’s gate electrode was the sensing platform, and DNA capture probes were immobilized on it via the APTES-GA method (Lin C. H. et al., 2022; Zhou et al., 2021; Huang et al., 2013b). The CMOS ISFET biosensor demonstrated HBV DNA detection in the range of 1 pM–10 nM and showed 53 mV/pH sensitivity under 100 MHz frequency.

Another ISFET-based biosensor for DNA hybridization detection was proposed by Chang et al. (Chang and Lu, 2020). The ISFET had a planar extended IDE and an external Ag/AgCl RE was used. The APTES-GA technique was employed to immobilize 5′– aminomodified HBV probe DNAs directly on the inter-metal ${SiO}_{2}$ surface which was thinned in a dry etching process to improve sensitivity. Solutions with DNA concentrations of 1 aM–1 μ M were tested with this biosensor, and shifts in the ISFET’s threshold voltage were observed with respect to DNA concentrations. The device showed a sensitivity of 32 mV/pH and an LOD of 1 fM.

In another work, Saengdee et al. developed an ISFET-based biosensor for urinary albumin determination, leveraging the ${Si}_{3}{Ni}_{4}$ passivation layer as the sensing membrane (Saengdee et al., 2021). An Ag/AgCl electrode was used as RE and anti-HSA was covalently linked to the ${Si}_{3}{Ni}_{4}$ through the APTES-GA method (Figure 7B). The drain current of the ISFET was kept constant, and the gate potential was monitored during HSA and anti-HSA interactions. A sensitivity close to the conventional immunoturbidimetry (IT) method was achieved in the range of 5–500 μ g/mL.

Zhang et al. achieved 58 mV/pH sensitivity with a novel three-dimensional-extended-metal-gate ISFET (3D-EMG-ISFET) (Zhang et al., 2019). The ISFET had a vertically extended gate fabricated by stacked metal layers and vias. The passivation layer ( ${SiO}_{2}$ and ${Si}_{3}{Ni}_{4}$ ) sitting above the top metal layer (Al) was opened using a reactive ion etching (RIE) process. The Al was exposed to air and created ${Al}_{2}O_{3}$ . This layer functioned as the pH sensing membrane and was further functionalized with ion-selective membranes (ISM) to detect ${Na}^{+}$ , $K^{+}$ , and ${Ca}^{+}$ . Specific ion receptors (ionophores) were embedded in polyvinyl chloride (PVC)/bis (2–ethylhexyl) sebacate (DOS) and dropped on top of 3D-EMG-ISFETs. For analyte detection, a constant voltage was applied between the FET’s drain and its source, and the solution was biased via an Ag/AgCl RE. pH and ion concentrations were retrieved from $I_{D}$ – $V_{G}$ plots, and sensitivities of − 57 mV/dec ( ${Na}^{+}$ ), − 48 mV/dec ( $K^{+}$ ), and − 26 mV/dec ( ${Ca}^{+}$ ) were reported.

##### 2.4.1.1 Biosensing with extended-gate ISFETs

When the analyte solution is in direct contact with the gate dielectric, any chemical reactions that occur in the fluid may affect the gate’s electrical properties and consequently modulate the ISFET’s $I_{D}$ – $V_{G}$ characteristics. This degrades the sensitivity of the biosensor, as the drain current is the primary figure of merit in ISFET-based biosensors. To overcome this issue (van der Spiegel et al., 1983) proposed an extended-gate ISFET (EG-FET) in which the sensing membrane is separated from the rest of the FET transducer. This gives the sensor greater immunity against environmental factors.

In a recent work, Kuo et al., (2021) successfully detected uric acid (UA) with 0.082 mg/dL LOD and 12.69 mV/(mg/dL) sensitivity with an extended-gate ISFET. The extended gate consisted of a ${RuO}_{2}$ sensing membrane that was fabricated on a PET substrate together with a custom-designed RE. Uricase was immobilized on ${RuO}_{2}$ using the APTES-GA method, and the extended gate was wired to the MOSFET gate and immersed in the solution. To detect UA, $I_{D}$ – $V_{G}$ and $I_{D}$ – $V_{D}$ curves were retrieved for different concentrations. It was observed that the curves varied for different concentrations due to the change in $V_{th}$ upon uricase-UA interactions.

**FIGURE 8:**
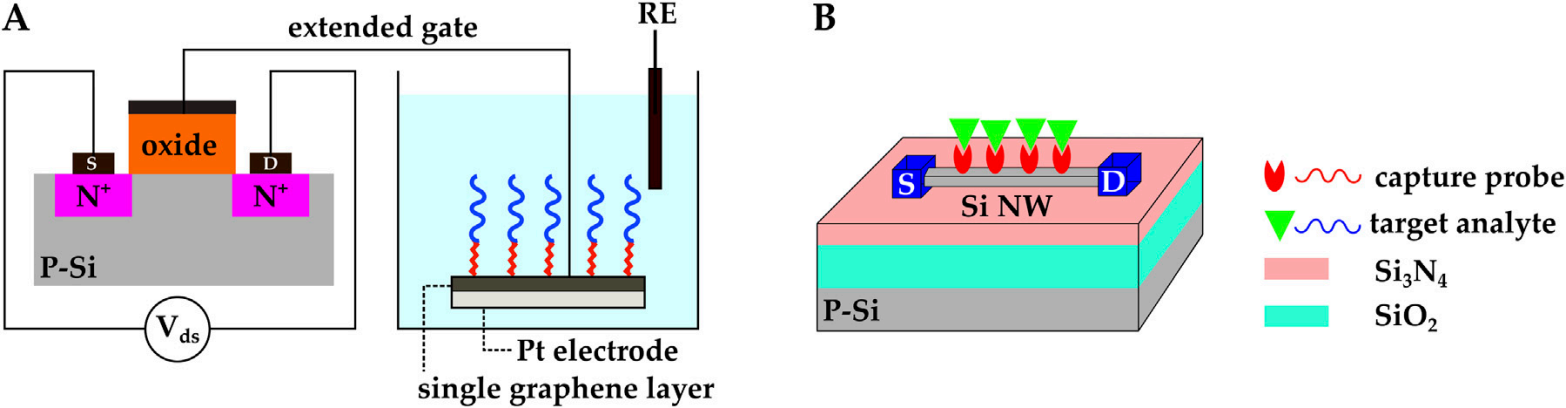
**(A)** EG-FET biosensor introduced by (Sheibani et al., 2021). The extended gate was a single-layer graphene on a platinum electrode. and it was connected to the MOSFET’s metal gate with metal vias. The extended gate and the Ag/AgCl reference electrode were exposed to the analyte solution, while the MOSFET transducer was isolated from the liquid under testing. Cortisol aptamers were immobilized on the graphene. **(B)** Schematic representation of a Si NW FET. The Si NW replaces the gate and the doped channel in regular MOSFETs, and the NW surface functions as the sensing membrane. Molecular probes are commonly immobilized on the NW’s surface.

Sheibani et al. used a novel EG-FET to develop a wearable cortisol hormone detector from human sweat (Sheibani et al., 2021). In this work, the extended gate was an aptamer-functionalized single-layer graphene on a platinum substrate. The aptamer was linked to graphene by the 1–pyrenebutyric acid N–hydroxysuccinimide ester (PBSE) linker molecule. The extended gate and an Ag/AgCl reference electrode were exposed to the analyte solution, while the MOSFET transducer was isolated from the liquid under test (Figure 8A).

#### 2.4.2 Biosensing with Si nanowire field-effect transistors

The introduction of 1D Si nanowires (NWs) goes back to the 1990s when single-crystal Si NWs were synthesized through vapor-liquid-solid (VLS) and laser ablation methods (Lieber, 1998). A CMOS-compatible top-down method for the fabrication of Si NW was introduced later in the early 2000s (Elibol et al., 2003) and made it possible to develop CMOS Si NWFETs. Due to NW’s large surface area, NWFETs can potentially offer higher sensitivities compared to regular FETs. NWs replace the gate and the doped channel in regular MOSFETs, and the NW’s surface functions as the sensing membrane. The surface of NWs is commonly modified by covalent immobilization of molecular probes (Figure 8B). Upon absorption of the target biomolecules, the charge distribution around the NW changes. The NW’s conductivity gets modulated, and the analyte’s presence is confirmed by this modulation (Syu et al., 2018; Ambhorkar et al., 2018; Mirsian et al., 2019; Ivanov et al., 2021).

One of the major challenges in developing NWFET biosensors is to selectively functionalize the NW’s surface and to avoid functionalizing the ${SiO}_{2}$ layer on the substrate. Mirsian et al. proposed modifications to the commonly used APTES-GA immobilization method to address this issue (Mirsian et al., 2019). They optimized the silanization reaction conditions, including oxygen plasma, APTES concentration and solvent, and silanization reaction temperature, so that the reaction happens only on the NW surface. Their results showed three times higher sensitivity compared to the non-selectively functionalized NWFETs.

Yong et al. developed a polycrystalline Si NWFET (pSiNWFET) biosensor for HBV detection (Yong et al., 2021) (Figure 8B). The HBV surface antigen (HBsAg) and the HBV X protein (HBX) were successfully detected in the 5.61 fM — 0.56 pM and 3.92 fM – 0.39 pM ranges, respectively. The pSiNWFET structure consisted of a silicon substrate as the back-gate electrode, a stacked silicon oxide/silicon nitride layer as the gate dielectric, two highly doped Si areas to create source and drain, and two polysilicon NWs to form the conducting channels. HBV antibody (HBsAb) and anti-HBX were immobilized on the pSiNWFETs surface with APTES and GA. The pSiNWFETs $I_{D}$ – $V_{G}$ curves were retrieved for different target analyte concentrations and shifts in the pSiNWFETs threshold voltages ( $\Delta \;V_{th}$ ) were observed as a function of concentration.

Krivitsky et al. designed a Si NWFET device for real-time monitoring of cancer cell’s metabolic activity and evaluating chemotherapeutic treatments (Krivitsky et al., 2019). The NWFETs surface was made sensitive to $H_{2}O_{2}$ –a byproduct of metabolic reactions–by chemical modifications. The 9,10–dihydroxyanthracene/9,10– anthraquinone (DHA/AQ) reversible redox molecular system was immobilized on the NWFETs surface, and the sensor was coupled to a microfluidic device that was functionalized with proper oxidase enzymes. The target metabolites were detected through sensing the $H_{2}O_{2}$ produced by the metabolic reaction. The limit of detection for $H_{2}O_{2}$ sensing was reported to be 100 nM.

In a recent work, a Si NW Schottky-junction FET (SiNW SJ-FETs) was introduced to detect DA in femtomolar concentrations. In a novel fabrication method, Si NWs were synthesized separately through a VLS process and later integrated into the CMOS chip by dielectrophoresis (DEP). Metal- ${NiSi}_{2}$ /intrinsic-silicon nano-Schottky junctions were formed within the nanowires and the sensor was covered by a layer of ${HfO}_{2}$ for passivation. The passivation layer was functionalized with DA DNA aptamers. The SiNW SJ-FET threshold voltage shifted in the presence of DA and a sensitivity of ∼ 1 V/fM was achieved (Sessi et al., 2022).

## 3 CMOS optical biosensing

In many biomedical and environmental applications, the presence of a specific molecule or nucleic acid sequence is detected through optical sensing. In optical biosensing, the illuminated element moves to an excited state and, upon relaxation, the excited electrons radiate their additional energy in the form of light. An image sensor captures the emitted light and translates it into an electrical signal, and information about the target can be retrieved from this electrical signal. Compared to charge-coupled devices, CMOS image sensors offer lower power consumption, monolithic integration of the pixel array and the auxiliary electronics, and higher temporal resolution. CMOS optical biosensors employ CMOS photodetectors as the light-to-electric signal converter, allowing the integration of the transducer and the front-end circuit on one chip to build compact and cost-effective devices (Forouhi and Ghafar-Zadeh, 2020; Sander et al., 2007). Moreover, they offer great advantages, such as enabling direct, real-time, and label-free detection of many biological and chemical substances while providing high specificity and sensitivity (Damborský et al., 2016; Lin C.-Y. et al., 2022).

**FIGURE 9:**
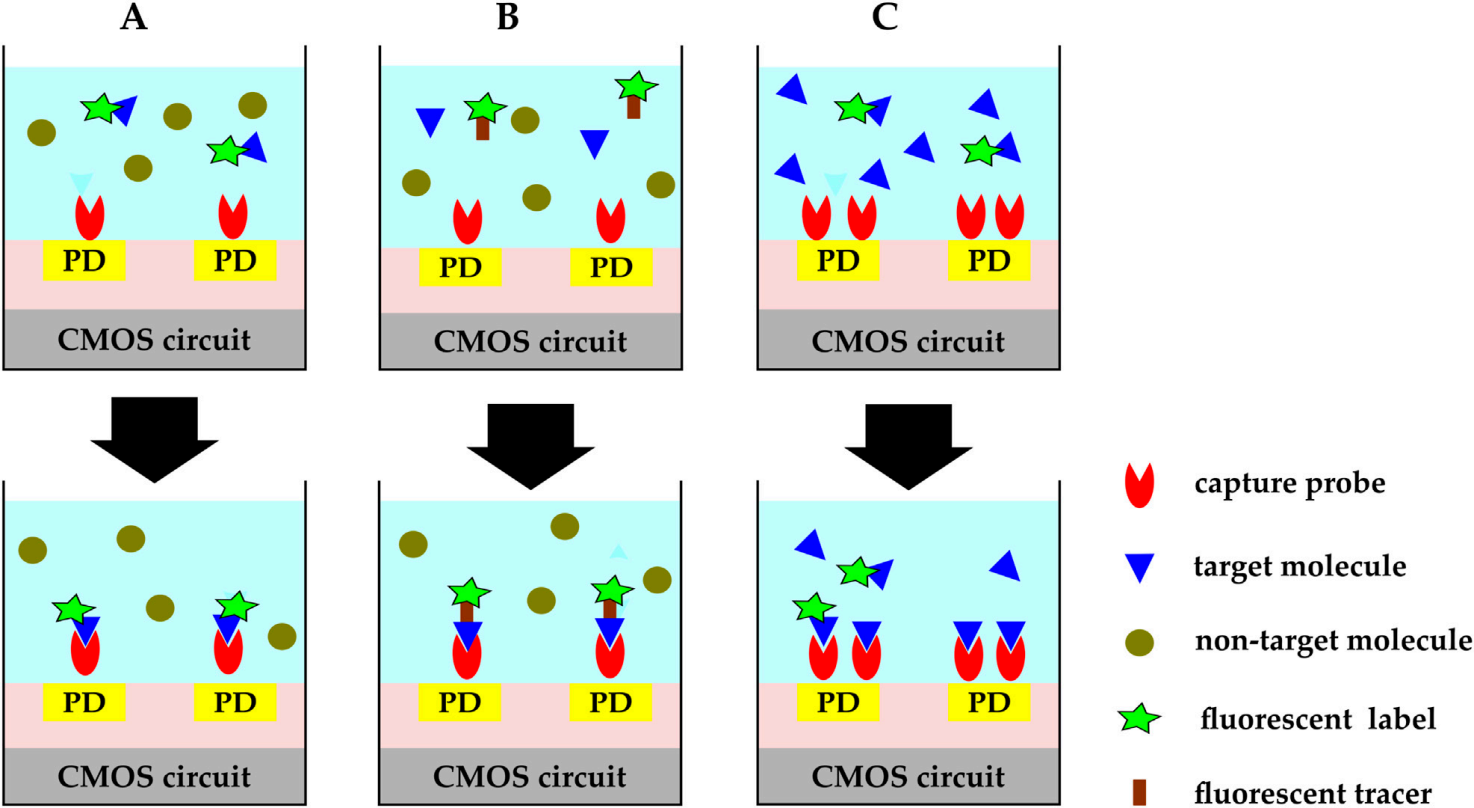
CMOS optical biosensing binding mechanisms:**(A)** direct. Capture probes are immobilized on the CMOS chip’s surface and target molecules are either inherently fluorescent or pre-labeled with a fluorescent group. **(B)** sandwich. Capture probes are immobilized on the sensor’s active region and fluorescent tracer molecules are added to the analytic solution. Target molecules bind to the capture probe and the fluorescent tracer molecule at the same time. Free fluorescent tracer molecules are physically removed from the solution or optically excluded from the sensing region. The remaining fluorescent signal is an indicator of the target biomolecule. **(C)** competitive. Capture probes are immobilized on the chip’s surface and pre-labeled target molecules are added to the sample solution while the target molecules in the analytic solution are not labeled. The added labeled molecules compete with the unlabeled molecules over the available binding sites. The fluorescent signal decreases as the concentration of the target molecules in the sample under test increases.

In optical biosensing, labeled or label-free analytes bind to the immobilized probes upon biorecognition events and induce changes in the optical field around the sensor. Optical biosensors employ four different mechanisms for detecting target analyte-BRE binding events: direct (Figure 9A), sandwich (Figure 9B), competition (Figure 9C), and displacement (Strianese et al., 2012). In direct binding, two species are involved. They are the capture probes, immobilized on the CMOS chip surface, and the target molecules. This approach can be used if the target analyte is inherently fluorescent or is pre-labeled with a fluorescent group. The sandwich assay is used when targets have at least two binding sites to bind to the capture probe and the fluorescent tracer molecule at the same time. Capture probes are commonly immobilized on the sensor’s active region, and fluorescent tracer molecules are added to the analytic solution. After the binding event, free fluorescent tracer molecules are physically removed from the solution or optically excluded from the sensing region, so the remaining fluorescent signal is an indicator of the target biomolecule.

Direct and sandwich binding are the two most widely used binding mechanisms; however, competitive or displacement assays are more widespread for small molecules with limited binding sites. In the competitive assay technique (McCall et al., 2012), capture molecules are immobilized on the chip’s surface and labeled targets are added to the sample solution, while unlabeled target molecules already exist in the analytic solution. The added labeled molecules compete with the unlabeled molecules over the binding sites, and the fluorescent signal decreases as the concentration of the target molecules in the sample under test increases. A slightly different mechanism takes place in the displacement method explained in (Wicks and Hargrove, 2019). In this displacement technique, labeled capture molecules are immobilized on the active sites and the biosensor is exposed to pulses of samples containing the target molecules. The target molecules wash away the fluorescent labels from the upstream zone, increasing the signal level at the downstream.

Optical biosensing is commonly classified into different categories based on their source of excitation. Fluorescence (external source), chemiluminescence (chemical reaction), electrochemiluminescence (electric potential), bioluminescence (biological reaction), and thermoluminescence (heat) are some of the major categories (Anand et al., 2022). In Sections 3.1, 3.2, we focus on the two most widely used techniques, fluorescence and chemiluminescence, and we briefly touch on two other optical detection mechanisms in Section 3.3.

### 3.1 Fluorescence biosensing

In fluorescence biosensing the fluorescent molecules need an external excitation source; for example, a light-emitting diode (LED) or a laser. The excitation source’s radiation power is much higher than that of the emission signals, and developing optical filters that integrate with CMOS biosensors to efficiently reject excitation photons from the emission signal is a major challenge (Dandin et al., 2007; 2012). To overcome this challenge, Hong L. et al. (2015, 2017) designed a fully integrated optical system-on-chip by exploiting optical interaction with sub-wavelength metal nanostructures made of electrical interconnect layers. Instead of constructing bulky optical filters on the chip’s surface, they used the metal layers available in the CMOS technology to integrate nanoplasmonic waveguide-based filters which were capable of more than 50 dB of rejection ratio across a wide range of incident angles. The PD glass surface was functionalized with capture probes and the streptavidin-coated Qdot 800 solution was used as fluorescent tags. This surface modification allowed the detection of 48 zmol of quantum dots, reaching a fluorescence/excitation ratio of nearly −62 dB without any post-CMOS fabrication, external optical filters, lenses, or collimators.

Another key challenge in CMOS fluorescence biosensing is exposing the CMOS chips to wet processes such as DNA and protein arrays immobilization. Surface functionalization is crucial to enhance specific binding and prevent adsorption of nonspecific analytes. Stadler et al. introduced an innovative surface coating for CMOS microchips utilizing poly (ethylene glycol)methacrylate graft polymer films *via* silanization (Stadler et al., 2007). The polymer layer provided high loadings of functional groups for probe molecules (anti-HA and anti-FLAG) immobilization. It also completely blocked nonspecific adsorption of proteins on the chip surface.

To enable highly parallel detection, the authors of (Hassibi et al., 2017; Manickam et al., 2017) created a fully integrated CMOS biochip that used an inverse fluorophore assay–previously described in (Hassibi et al., 2009)–to perform parallel polymerase chain reaction (PCR). The biochip consisted of a 32×32 biosensing microarray, each pixel being a 100×100 $\mu m^{2}$ biosensor that contained a CMOS photosensor and a resistive heater. Additionally, the platform was improved with a multi–dielectric interference filter and silanization was used to covalently bond fluorogenic DNA capture probes to the chip’s ${SiO}_{2}$ surface. The biochip was able to continuously detect FluA, FluB, RSV, PIV, AdvE, AdvC2, and polio without requiring any labels or sandwich probes. Recently (Manickam et al., 2021), demonstrated the detection of SARS-CoV–2 in addition to the previously detected upper respiratory pathogens. The immobilized DNA capture probes on the sensor chip and performed time-gated, bio-luminescence, and continuous-wave fluorescence detection. The photodiode exhibited a > 130 dB detection dynamic range over a 25–100 ° C temperature range with a heating and cooling rate of >±10° C/s.

In a recent work (Zhu et al., 2022), created a fluorescent CMOS biosensor that can be ingested for monitoring the gastrointestinal (GI) tract. The biosensor was a single CMOS chip that integrated a 15-pixel fluorescence sensor array, a low-power wireless interface, and on-chip nanoplasmonic optical filters. All sensing and processing electronics were enclosed within a 1.2 cm × 2.5 cm biocompatible casing and the Tx/Rx (transmitter/receiver) antennas were linked to the CMOS chip. The biosensor demonstrated pM sensitivity to the biomolecules present in the GI tract.

### 3.2 Chemiluminescence biosensing

Unlike fluorescence, chemiluminescence does not require an external excitation source, and the excitation energy is provided by the chemical reactions involved in the biological events. Not having a strong background illumination noise, chemiluminescence can have better sensitivity compared to fluorescence-based assays (Redy Keisar et al., 2024). It is also selective and enables multi-analyte parallel detection. Moreover, since light is produced inherently, no filter is needed (Selvam, 2017), which allows more compact, miniaturized, and low-cost LOC designs (Kricka, 1995). Chemiluminescence’s disadvantage compared to fluorescence measurement techniques is its higher susceptibility to changes under environmental conditions (Tokuda and Ohta, 2022).

Chemiluminescence detection methods are usually named after their luminescent-producing reagent. Luminol- and peroxyoxalate-based chemiluminescence are two of the most widely used chemiluminescence sensing techniques (Poupon-Fleuret et al., 1996; Sigvardson et al., 1984). Luminol exhibits chemiluminescence with a blue glow upon oxidation. Peroxyoxalates is an intermediate, and it transforms into 1,2–dioxetanedione. The 1,2–dioxetanedione compound decomposes into carbon dioxide ( ${CO}_{2}$ ) and shows chemiluminescence in the presence of chemiexcitation, and photodetectors record the optical signals. Sometimes, the optical signals can be weak and fast-decaying, requiring a highly sensitive time-resolved detector.

To improve sensitivity (Sandeau et al., 2015), presented a large-area (150 μm×150 μm) and highly sensitive CMOS biopixel array of 182 pixels capable of compact multiplex biosensing. The system utilized supercritical angle luminescence (SAL) (Enderlein et al., 1999) to improve the detection sensitivity of Tumor Necrosis Factor-alpha (TNF- α ), Interferon-gamma (IFN-γ), and Interleukin-8 (IL-8), while keeping the device simple enough for LoC application. The large format of the pixel accommodates standardized liquid spotting and biofunctionalization methodologies, circumventing sample spot overlapping and facilitating parallel detection of the three targets. To increase light capture efficiency, the authors functionalized the silicon substrate with antibody, instead of the glass passivation layer. They performed silanization on the surface of the biopixel arrays with (3- glycidyloxypropyl)trimethoxysilane (GOPTS), to enable covalent bonding of capture antibodies, following an $O_{2}$ -plasma activation. Capture antibody solutions were prepared in phosphate buffered saline (PBS) with glycerol, IFN, IL-8 and TNF- α . They utilized the high refractive index of silicon to enable SAL. This resulted in a 100 fold improvement in sensitivity compared to the conventional chemiluminescence systems.

Another study by (Joung et al., 2012) demonstrated the utility of chemiluminescence to detect human interleukin 5 (IL-5) of sub- pg/ml concentration by developing a more sensitive platform. While CMOS imagers find typical use cases as detectors in chemiluminescence setups, the authors used them as a detector and substrate for functionalization. To be specific, IL-5 capture antibody was immobilized on the surface of the image sensor and bovine serum albumin (BSA) was used for blocking to suppress non-specific bindings. Finally, IL-5, biotin-labeled detection antibody, and streptavidin-conjugated horseradish peroxidase were reacted in sequence to form the sandwich immunoassay. In addition to functionalizing the sensor surface with the sandwich format, they employed pixel counting analysis for better luminescence signal detection. The detection sensitivity obtained for IL-5 was evaluated up to 0.1 pg/mL .

Another study (Hong D. et al., 2015) demonstrated further improvements in sensitivity to attomolar level detection through direct antibody immobilization on the surface of the CMOS image sensor, as well as using a simple data accumulation process with noise calibration. The chip surface was modified to introduce aldehyde groups using GA and the capture antibody was applied to the surface of the chip and incubated at 4°C overnight. The chip showed that the logarithmic linear range for interleukin-5 (IL-5) detection is 1 fg/mL to 20 ng/mL and the limit of detection is 0.074 fg/mL . Furthermore, multiple target detection including IL-2, IL-4, IL-5, and IL-6 was accomplished simultaneously with their low-cost array type CMOS image sensor.

Al-Rawhani et al. developed a quadruple-mode CMOS biosensor for rapid biomarker detection. The sensor had colorimetric, chemiluminescent, surface plasmon resonance (SPR), and hydrogen ion measurement modes (Al-Rawhani et al., 2020). The CMOS chips included an array of 16×16 pixels. Each pixel contained a PD, a single-photon avalanche diode (SPAD), and an ISFET. The SPAD performs well in chemiluminescence detection, because no external excitation source is involved and the risk of saturation is low, while a highly sensitive photo detector is needed (Pires et al., 2014; Ingle et al., 2019). Gold nanodiscs were fabricated on top of the PDs and SPADs in post-CMOS processes to facilitate SPR. Uric acid detection was demonstrated via chemiluminescence. This multimodal biosensor is discussed in more detail in Section 4.4.

### 3.3 Beyond fluorescence and chemiluminescence biosensing

The absorption-based optical biosensor is a viable optical detection platform that relies on photon absorption instead of photon emission. Hofmann et al. examined a CMOS biochip photometer in which 100 μ m ×100 μ m pixel sensors were arranged in a 6×7 pixel array for prostrate specific antigen (PSA) detection (Hofmann et al., 2020; 2021). Capture antibodies were immobilized on the CMOS chip surface. The biosensor showed a limit of detection of 0.5 ng/mL within 6 min.

Liu et al. developed an ingestible 6.5 mm × 12 mm capsule benefiting from bio-luminescence (Liu et al., 2022). They successfully showcased the detection of low-intensity bioluminescent signals produced by bioengineered bacterial sensors upon exposure to the intestinal inflammation biomarker, tetrathionate (*in vitro*). Four channels of CMOS PDs (p+/nwell/psub) were exposed to four chambers containing genetically engineered bacteria.

## 4 Beyond electrochemical and optical CMOS biosensing

Electrochemical and optical biosensors form the bulk of developed CMOS biosensors. However, compelling novel CMOS biosensing devices have been designed that employ other sensing mechanisms, namely, electrical, mechanical, and magnetic transduction. In this section, we review some of the recent works in CMOS biosensing in these areas.

### 4.1 Electrical biosensing

There is a very fine line between electrochemical and electrical transducing methods. Electrical biosensors are “devices based on measurements where no electrochemical processes take place, but the signal arises from the change of electrical properties caused by the interaction of the analytes (Hulanicki et al., 1991); ” while electrochemical biosensors transform the electrochemical effect of the analyte-electrode interaction into a measurable signal. This distinction underscores the nuanced differences between the two types of sensors in their signal generation mechanisms.

**FIGURE 10:**
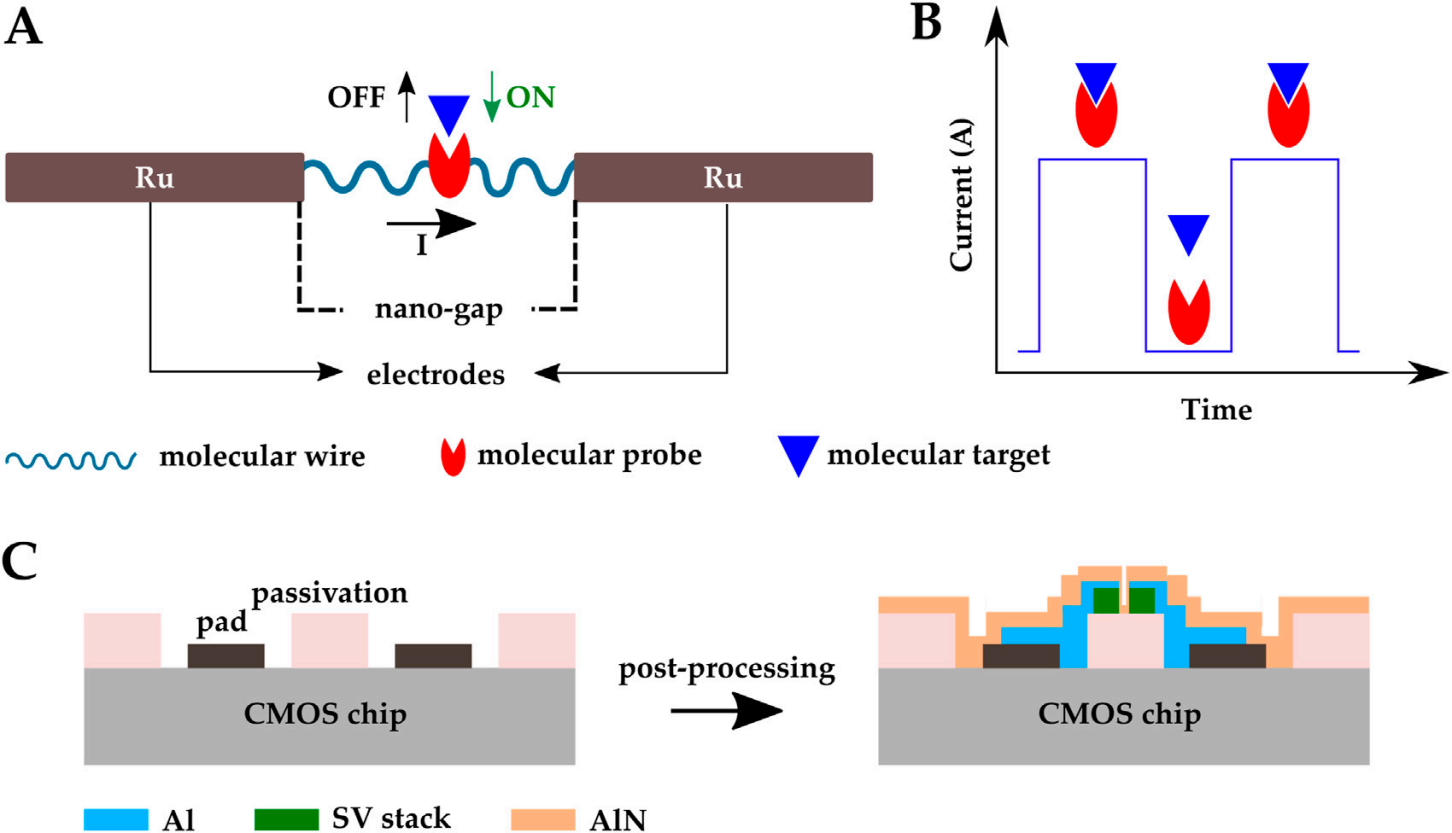
Schematic representation of the CMOS electrical biosensor developed by (Hall et al., 2022); **(A)** a peptide molecular wire connecting two Ru nanoelectrodes, **(B)** a series of current pulses indicating binding events happening. **(C)** CMOS magnetic biosensor developed by (Costa et al., 2017); SV sensors were post-fabricated on top of a CMOS IC front-end and the sensors were covered by an AlN passivation layer.

A CMOS molecular electronic chip was presented in (Hall et al., 2022) for single-molecule biosensing. Aviram et al. first used single molecules as circuit elements (Aviram and Ratner, 1974). Using this concept, Hall et al. developed a CMOS biosensor to detect DNAs, proteins, enzymes, and antibodies (Hall et al., 2022). The primary sensing element was a molecular peptide wire connecting two ruthenium (Ru) nanoelectrodes. The two nanoelectrodes had a gap of less than 30 nm and were fabricated on a 16k pixel CMOS current reader chip in post-CMOS fabrication steps. The peptide wires were loaded on each pixel and attached to the nanoelectrodes via dielectrophoresis (DEP). The Probe molecules were positioned in precisely specified sites on the wire (Figure 10A). The nominal resistance for a 25 nm long alpha-helical peptide molecular is ∼ 100 G Ω . When a binding event occurs, the sensor impedance changes, resulting in a change in the current that passes through the wire. Since the binding events are reversible, the current is monitored transiently and a series of current pulses indicates binding events happening (Figure 10B). Direct real-time detection of single-molecule interactions was achieved.

### 4.2 Magnetic biosensing

Biological sample metrics like blood, urine, etc are non-magnetic. Unlike electrochemical biosensors that are heavily affected by pH, ionic strength, and the temperature of their surrounding environment, magnetic sensing offers a matrix-insensitive biomarker detection platform. Immunity from matrix interference enhances the sensitivity and selectivity of the system (Zhou et al., 2021). Wang et al. and Sun et al. reported magnetic sensing with sub-ppm sensitivities (Wang et al., 2014; Sun et al., 2022). Magnetic biosensors can be based on a variation of transducers such as the Hall-effect sensor (Gambini et al., 2013; Lei et al., 2017), LC oscillator (Wang et al., 2014; Sun et al., 2022), or magneto resistive (MR) sensor (Han et al., 2006; Zhou et al., 2021; Hall et al., 2013; Costa et al., 2017).

Among magnetic sensors, giant magneto resistive spin valves (GMR SV) are CMOS compatible. These sensors are great candidates for LOC development and mass production (Adem et al., 2020). Costa et al. fabricated SV sensors on top of a CMOS IC front-end and covered the sensors with an AlN passivation layer (Figure 10C) (Costa et al., 2017). SV sensor’s fabrication included multiple steps of photo-lithography, electron beam deposition, sputtering, lift-off, and ion milling etching. The AlN passivation layer of the SV sensor array can be functionalized with biological probes for biomolecular recognition. The system achieved a magneto resistive ratio of 5.37% and successfully detected 250 nm magnetic nanoparticles.

### 4.3 Mechanical biosensing

Advances in microelectromechanical systems (MEMS) since the 1980s have facilitated the development of mechanical transducers. Microcantilever-based biosensors detect biological events by monitoring small displacements of the cantilever. The biosensor works by immobilizing a BRE on the cantilever surface, which captures target molecules and induces changes in the cantilever’s bent or resonance frequency. Microcantilever-based biosensors offer high sensitivity, fast response, and portability (Lavrik et al., 2004; Huang et al., 2013a; Basu et al., 2020; Li et al., 2008).

**FIGURE 11:**
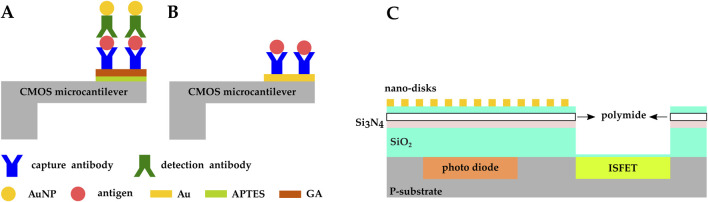
CMOS microcantilever-based biosensors: **(A)** the cantilever’s surface was oxidized and AFP capture antibodies were covalently immobilized on it *via* APTES-GA crosslinking. AuNPs were functionalized with detection antibodies (Zhao et al., 2021); **(B)** Vp capture antibodies were immobilized on the Au electrodes fabricated on the CMOS microcantilever (Wang et al., 2021). **(C)** CMOS multimodal biosensor developed by (Al-Rawhani et al., 2020).

Zhao et al. demonstrated a CMOS microcantilever biosensor to detect alpha fetoprotein (AFP), a protein produced by the liver and in the yolk sac of a fetus (Zhao et al., 2021). The Si cantilever’s surface was oxidized and the AFP capture antibodies were covalently immobilized on it via APTES-GA crosslinking, while gold nanoparticles (AuNP) were functionalized with detection antibodies (Figure 11A). Detection antibodies bound to the antigen, and the antigen bound to the capture antibody immobilized on the sensor surface. This brought AuNPs in closer proximity to the sensor and induced detectable shifts in the cantilever resonance frequency. The sensor was able to detect AFP with a limit of detection (LOD) of 21 pg/mL and a linear range of 0–70 ng/mL.

In another study by the same group, a CMOS microcantilever biosensor was developed based on an alternating current electrothermal technology (ACET) configured to detect bacteria. Gold electrodes were fabricated on the CMOS microcantilever and functionalized with mercaptan self-assembled monolayer (SAM) layer, and *Vibrio* parahaemolyticus (Vp) capture antibodies were immobilized on the functionalized Au-patterned Si cantilever (Figure 11B). The ACE effect improved molecular aggregation around the microcantilever which led to an increase in binding probability, resulting in an LOD of $5\times 10^{5}$ CFU/mL, 10 times lower than its counterpart without ACET. The biosensor showed linearity in the × $10^{5}$ – $10^{7}$ CFU/mL range (Wang et al., 2021).

### 4.4 Multimodal biosensing

Monitoring cellular processes is essential in diagnostic and therapeutic research and applications. Although many cell-based biosensors (CBBs) have been developed to study cellular activities, for example, cell proliferation (Weeks et al., 2022), apoptotic cell death (Akçapınar et al., 2021; Garcia-Hernando et al., 2020), and cytotoxicity (Garcia-Hernando et al., 2020), most of the CBBs developed use only one transduction mechanism and are capable of monitoring one kind of biophysiological response (Wang et al., 2022a), while cellular activities are inherently diverse in type and scale (Hu et al., 2021). As CMOS technology allows integration of multiple sensors on one chip to monitor different types of signals (i.e., electrochemical, optical, etc.), multimodal CMOS CBBs have been introduced to capture multi-physiological cellular responses (Tokuda et al., 2006; Hu et al., 2021; Jung et al., 2021; Kumashi et al., 2021; Wang et al., 2022a).

Hu et al. introduced a multimodal CMOS biosensor array consisting of 131,072 pixels with three $N_{\mathrm{well}}$ – $P_{\mathrm{sub}}$ photodiodes (PD), one NMOS ISFET, and one impedance measurement circuit integrated in each pixel to perform color-sensitive imaging, pH detection, and EIS measurement (Hu et al., 2021). To enable pH sensing and EIS, the top metal layer (Al) was chemically etched away from the ISFET area, exposing the underlying titanium nitride (TiN) layer as the working electrode, and an external Ag/AgCl reference electrode was used. Cultured bacteria colonies were directly placed on the sensor array and imaged through EIS. The PD peak sensitivities were 11 mA/W at 700 nm and 195 mA/W at 525 nm and the measured pH sensitivity was 27.7 mV/pH.

Wang’s group recently reported multiple multimodal CMOS biosensors to study cellular processes. For example (Kumashi et al., 2021), performed both amperometric and impedimetric electrochemical biosensing on a 256-pixel CMOS sensor array to characterize exoelectrogens. The chip had 2×256 WEs, 16 REs, and 32 CEs. The original Al electrodes of the CMOS chip needed to be replaced with biocompatible inert electrodes through post-CMOS fabrication steps. For this purpose, the Al electrodes were etched away and gold electrodes were deposited to replace them. The gold electrodes served as WEs and CEs. REs were formed by depositing three additional layers on the Au layer: Ag/AgCl/PEDOT:PSS. Exoelectrogenic *Shewanella oneidensis* MR–1 bacteria were successfully detected by amperometry with a current sensitivity of 0.204 pA. Furthermore, HEK–293 cancer cells were cultured on chip and their adhesion to the electrode surface was successfully captured by impedance measurements.

The same group developed another multimodal CMOS CBB (Jung et al., 2021). In this biosensor, PDs and Au electrodes were integrated in a 21,952-pixel CMOS chip for potential, optical, and four-point impedance sensing. Au electrodes were fabricated using the same method described in (Kumashi et al., 2021). Holistic on-chip cell characterization was performed on rat cardiomyocytes and single-cell resolution was achieved.

Wang et al. employed the same method as (Kumashi et al., 2021; Jung et al., 2021) to fabricate biocompatible on-chip electrodes, except they used platinum instead of gold (Wang et al., 2022b; a). Pt has lower electrode-electrolyte impedance and is a better option for impedance sensing applications. Human stem cell-derived neurogenin 2–accelerated progenitor cells (SNaPs) and mouse skeletal myoblast differentiated into muscle cells (C2C12) were encapsulated in on-chip cultured brain modeling hydrogels. Electrical tests and biological measurements successfully demonstrated multimodal CBB (Wang et al., 2022b).

(Al-Rawhani et al., 2020) developed a CMOS biosensor for rapid biomarker detection. The sensor had four modes of detection: colorimetric, chemiluminescent, surface plasmon resonance (SPR), and hydrogen ion measurements. The CMOS chip had an array of 16×16 pixels and each pixel contained three transducers: a PD, a SPAD, and an ISFET. PDs were used for the colorimetric mode when an external illumination source was involved and the risk of saturation was higher while SPADs were used in detecting chemiluminece by detecting weak and fast-decaying signals. Au nano-discs were fabricated on top of PDs and SPADs in post-CMOS processes to exhibit SPR. Prior to Au nano-discs fabrication, a layer of ${SiO}_{2}$ was deposited on the sensor via PECVD. ISFET was exploited for pH sensing. The top Al layer was etched away via a wet, etch process to expose an additional ${SiO}_{2}$ layer on top of an ISFET floating gate (Figure 11C). This oxide layer functioned as the sensing membrane for ISFET and an external Ag/AgCl RE was used. Glucose, cholesterol, urea, and urate were successfully detected, all within their physiological ranges.

## 5 Surface modification and functionalization methods

Although CMOS technology offers many advantages for the development of biosensors, the interconnect metal available in CMOS technology, aluminum, is not suitable for biosensing applications because it is prone to corrosion and is not polarizable. Inert polarizable metals, such as gold and platinum, are not available in the standard CMOS processes. Additionally, sensor surface functionalization and probe immobilization, which play crucial roles in affinity-based detection applications, cannot be performed through CMOS technology. To address these issues, post-CMOS processes are used to fabricate biocompatible functionalized electrodes (Kuo et al., 2020). In this section, we review post-CMOS processes developed for fabricating functionalized electrodes on the CMOS chips. These processes have two main steps; the first step is fabricating the electrodes and the second step is to immobilize the capture probes on the electrodes.

### 5.1 Electrode fabrication

The common method to fabricate on-chip electrodes is to initially design the electrodes integrated in the CMOS chip using the Al top metal layer and to later modify the electrodes in the post-CMOS fabrication steps. This can be as simple as a one-step etching process. Zhang et al. and Tabrizi et al. removed the passivation layer on top of the Al electrodes via reactive ion etching (RIE) and chemical etching, respectively, to expose the Al electrodes (Zhang et al., 2019; Tabrizi et al., 2021). Once exposed to air, Al turns into ${Al}_{2}O_{3}$ which is a biocompatible oxide. Hu et al. chemically etched away the passivation and the top metal layer to expose the TiN electrodes underneath (Hu et al., 2021). Lin et al. and Lee et al. stuck with their Al electrodes and only dry etched the passivation layer on top of the electrodes, so the oxide is as thin as 110 nm and the Al electrodes sensitivity improves (Lin C. H. et al., 2022; Lee et al., 2021).

Polarizable metals, i.e., noble metals such as gold and platinum, are preferred for electrochemical sensing applications. In these cases, the post-CMOS process includes at least two steps; removing both the Al electrodes and the passivation layer on top of them and depositing noble metal electrodes (Au and Pt) instead. For example, Wang et al. chemically etched the Al electrodes and fabricated the Pt electrodes by sputtering (Wang et al., 2022a; b). Kumashi et al. and Jung et al. removed the Al electrodes by chemical etching and fabricated the Au electrodes using E-beam evaporation (Kumashi et al., 2021; Jung et al., 2021). Additionally, more complex processes have been developed, including multiple steps of photolithography, electron beam deposition, sputtering, lift-off, etching, DEP, PECVD, etc. Examples can be found in (Al-Rawhani et al., 2020; Hall et al., 2022; Sessi et al., 2022; Costa et al., 2017; Wang et al., 2021).

### 5.2 Probe immobilization

Various techniques have been studied and employed for probe immobilization, including physical adsorption, streptavidin-biotin interaction, and covalent immobilization. Physical adsorption relies on the ionic interactions between biomolecules and the transducer surface, and its main advantage is its simplicity. This method does not require linker molecules or target analyte modifications. The streptavidin-biotin complex exhibits one of the strongest noncovalent bonds. Immobilizing either streptavidin or biotin on the sensor surface and subsequently biotinylating biomolecules is a well-established technique for detecting proteins and DNA. Biotin, as a label, is known to be highly stable and has minimal impact on the function of the labeled molecule (Nimse et al., 2014; Mirsian et al., 2019; Stern et al., 2007; Hong L. et al., 2015, 2017).

**FIGURE 12:**
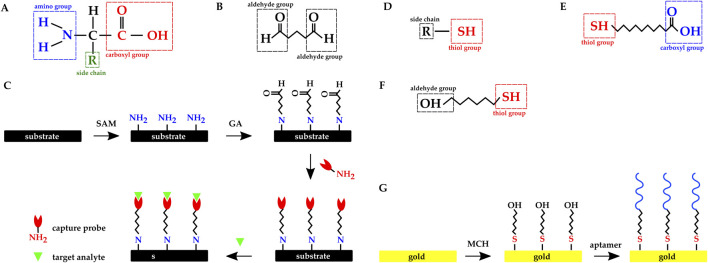
Chemical structure of **(A)** Amino acid. **(B)** Glutaraldehyde molecule. **(C)** Overview of the dielectric substrate preparation with SAM and GA molecules: amine functionalization, activation with glutaraldehyde, probe immobilization, and immunoassay. Chemical structure of **(D)** a molecule with thiol group. **(E)** 11-mercaptoundecanoic acid. **(F)** 6 mercapto 1 hexanol. **(G)** Overview of the gold electrode’s functionalization with MCH SAM and thiolated aptamer.

Among the immobilization methods mentioned above, covalent immobilization has the highest binding strength and stability. A popular functionalization technique is to leverage the amine group (– ${NH}_{2}$ ) present in amino acids (Figure 12A) and the crosslinker molecules to covalently immobilize both the capture and the target molecule on the biosensor’s surface. Amine has strong affinity with multiple functional groups such as carboxyl (–COOH), aldehyde (–COH), sulfonic (– ${SO}_{2}$ OH), epoxy (– $C_{2}H_{2}$ O) and isothiocyanate (–NCS). Amine affinity with these functional groups is employed for surface functionalizations. Crosslinkers are molecules with two binding sites and they bind to the amine present in the target molecule with one of their binding sites, and their second binding site is used to bind to an SAM.

Glutaraldehyde (GA) is a crosslinker molecule possessing two aldehyde (–COH) functional groups (Figure 12B). To leverage the crosslinking method, an SAM with amine functional group is introduced to the sensor’s (electrodes’) surface, and GA binds to the SAM with one of GA’s binding sites. The other binding site will be available to the capture probe to bind to. Finally, the target protein will covalently bind to the capture probe (Figure 12C). SAM is chosen based on the electrode’s surface material; for example, APTES has been frequently used to functionalize dielectric surfaces (Zhao et al., 2021; Lin C. H. et al., 2022; Lee et al., 2021; Chang and Lu, 2020; Saengdee et al., 2021; Yong et al., 2021).

Gold has a strong affinity for thiol (–SH) (Figure 12D) and the SAMs used in functionalizing gold electrodes commonly have this group. Zhao et al. functionalized their gold electrodes with antibodies using 11-mercaptoundecanoic acid (11-MUA) SAM (Figure 12E) and EDC crosslinker (Zhao et al., 2021). Singh et al. used six mercapto one hexanol (MCH) (Figure 12F) SAM to functionalize their Au IDEs with thiolated aptamers (Figure 12G) (Singh et al., 2019). Kuo et al. and Lee et al. directly immobilized thiol-modified DNAs and apatamers on gold electrodes without any SAM (Kuo et al., 2020; Lee et al., 2012).

## 6 Discussion and future trends

CMOS technology enables the integration of transducers and readout circuits in a single chip to build biosensors; moreover, the advances in the post-processing steps that are compatible with CMOS chips have made CMOS biosensors more versatile. In this work, we reviewed recently developed CMOS biosensors with surface modifications. These biosensors use different sensing modalities for a wide variety of applications, from detecting DNA (Manickam et al., 2019a), proteins (Mirsian et al., 2019), and enzymes (Andrianova et al., 2018) to monitoring cell viability (Senevirathna et al., 2018). Here, we highlight the significance of the reviewed devices, highlighting their advantages as well as their potential limitations.

Nasri et al. (2017) and Manickam et al. (2019a,b) developed amperometric biosensors for DA and DNA detection respectively. Each of these biosensors has its own advantages and limitations. For example, through multiple steps of post-processing, Nasri et al. integrated the WE, CE, and RE in the CMOS chip, while Manickam et al. integrated the WE and CE, and used an external RE. On the other hand, Manickam et al. functionalized the biosensor’s electrodes with complementary target DNA and examined the sensors specificity, whereas Nasri et al. did not functionalize their biosensor and there is no data available on measurements in the presence of other neurotransmitters, which leaves the sensor’s specificity untested. Doi et al. (2022) used a potentiometric approach to capture spatiotemporal dynamics of ATP. They fabricated gold electrodes on their CMOS potentiometric sensor and similar to Nasri et al. (2017), this biosensor needs and external RE. Having gold electrodes in addition to the need for an external RE, increases the biosensor’s cost; however, Doi et al. functionalized their biosensor using a mixed-layered technique based on physical adsorption which could make the biosensor potentially reusable; However, its re-usability was not explored.

Kuo et al. (2020) and Lin C. H. et al. (2022) developed capacitance biosensors for DNA detection and covalently immobilized complementary probe DNAs on the electrodes. Kuo et al. fabricated gold electrodes on their biosensor while Lin et al. simply thinned the passivation layer on top of the Al electrodes. Using gold electrodes enables improved LOD and sensitivity, but it complicates the post-CMOS processing and increases the manufacturing cost. Depending on whether cost or sensitivity is prioritized, these two approaches are interchangeable.

Another category of CMOS biosensors discussed is ISFET-based biosensors. Lee et al. (2021) and Chang and Lu (2020) thinned the passivation layer on top of the ISFET gate and covalently immobilized DNA probes on the remaining thinned passivation layer. Lee et al. studied the optimized operating frequency while Chang et al. explored different electrode sizes and shapes. The latter work reported better pH sensitivity and significantly lower LOD. A more in depth look into these two biosensor’s designs would give helpful information about the electrochemistry of the biosensor and the transduction mechanism. Saengdee et al. (2020) on the other hand did not perform any post-CMOS processing and they covalently immobilized the capture antibodies on the passivation layer. As discussed above, this approach simplifies the biosensor development process but it has a negative impact on the sensitivity. Kuo et al. (2020) and Sheibani et al. (2021) fabricated gates separately, functionalized them, and wire-connected them to the CMOS FETs for UA and cortisol detection, respectively. Having an extended gate improves the ISFET’s sensitivity as the FET stays immune from the changes in the analytic environment; moreover, it makes the functionalization process easier.

We discussed NWFET-based CMOS biosensors as well (Yong et al., 2021; Krivitsky et al., 2019; Sessi et al., 2022). Because of their larger surface area, NWFETs have the potential to achieve higher sensitivities compared to ISFETs; however, NWFETs have more complex fabrication processes. Consequently, they have not been explored as much as other CMOS electrochemical biosensors.

Hassibi et al. (2017) and Manickam et al. (2017) integrated optical filters and resistive heaters with CMOS photosensors to build a portable PCR machine with continuous fluorescence detection. Zhu et al. (2022) also used a CMOS photosensor with integrated filters to built an ingestive fluorescence sensor for GI monitoring. On the other hand, Sandeau et al. (2015) and Joung et al. (2012) built CMOS chemiluminescence biosensors without having to fabricate integrated filters. They functionalized their sensors’ surfaces with appropriate probes to detect cytokines like TNF- α and IFN-γ. Although fluorescence detection is a more common optical detection method, chemiluminescence detection approaches are more suitable for integration with CMOS sensors since they do not require optical filters.

We also discussed CMOS biosensors that use electrical, magnetic, and mechanical transducers. The electrical CMOS biosensor developed in (Hall et al., 2022) provides real-time single molecule detection; however, the sensor development process is very complicated. Costa et al. (2017) developed a magnetic biosensor by fabricating a spin-valve on a CMOS IC. Compared to the other types of biosensors discussed, the magnetic biosensor offers better matrix-insensitivity which enhances the biosensor’s sensitivity and specificity; nevertheless, the post-processing includes multiple steps of photo-lithography, electron beam deposition, sputtering, lift-off, and ion milling which makes the sensor development process costly. Zhao et al. (2021) and Wang et al. (2021) developed cantilever-based mechanical CMOS biosensors for bacteria detection by immobilizing antibody on the oxide and gold electrodes fabricated through post-processing steps. The cantilever-based biosensors offer faster response, yet the sensitivities reported in works reviewed did not exceed that of other biosensors with different sensing modalities.

CMOS technology offers integrating multiple transducers with different sensing modalities in one chip. Having multiple transducing mechanisms simultaneously enables multi-dimensional sensing; however it should be noted that it increases the CMOS sensor design and fabrication cost, as it requires more complicated circuitry as well as larger die area. Hu et al. (2021) and Kumashi et al. (2021) used this advantage to build CMOS biosensors for monitoring cultured bacteria activity. Hu et al. integrated three PDs and two ISFETs in each pixel and used an external RE to enable imaging, pH sensing and EIS. To improve sensitivity, they fully etched away the Al ISFET gate and used the TiN layer as ISFET sensing membrane. Kumashi et al. on the other hand, fabricated gold electrodes to integrate WE, CE, and RE on the CMOS chip and enable fully-integrated amperometric and impedimetric biosensing. Wang et al. (2022a) replaced gold electrodes with Pt since it has lower electrode-electrolyte impedance. Similar to the biosensors discussed earlier, these works have different approaches to electrode design, balancing sensitivity and cost.

The works we discussed above have made major contributions to the filed. However, there are still many challenges to be addressed in order to achieve the mass production of the functionalized CMOS biosensors. For example, although post-CMOS processes such as oxide etching and metal deposition contribute to the CMOS biosensor performance improvement, they require access to micro- and nano-fabrication facilities equipped with photo lithography, etching, and metal deposition tools. In addition, process engineers must optimize the fabrication steps for each device. This can be a costly and time-consuming process as they should consider factors such as the chips’ dimensions, their minimum feature size, the thickness and material of the passivation layer to be etched, and the metal to be deposited. Some CMOS foundries offer special post-CMOS processes compatible with noble metals, by exploiting which, in-house fabrication steps can be skipped to some extent; however, it should be noted that these processes are mostly in the experimental phase and hence costly.

The other step of modifying CMOS biosensors is functionalizing the chip’s surface with the bio-recognition elements. Covalent immobilization is a widely used method for functionalizing biosensors, because it is the most stable compared to other approaches such as physical adsorption; however, the disadvantage of covalent bonding is that these bonds need to be broken for purposes such as cleaning the chips and reusing them for other applications. In these cases, the chip has to be exposed to harsh environmental conditions such as high acidity or high temperature. This can decrease the sensor’s life-time significantly.

Another disadvantage of covalent bonding is that it depends on the chemical reactions occurring between the substrate material and the SAM molecules. So, to achieve spatially selective functionalization, the sensing surfaces need to be patterned with different materials, which makes post-CMOS processing even more complicated. This issue can be addressed by using polymer-based electroactive hydrogels such as chitosan instead of SAM to enable localized functionalization. Moreover, compared to covalently bound SAMs, hydrogels are easier to remove (Buckhout-White and Rubloff, 2009; Wu et al., 2003; Tseng et al., 2014; Susanto et al., 2013; Kim et al., 2015).

It is important to note that the mass production of CMOS biosensors that can compete with commercialized POC technologies, such as test strips, in cost, demands having a deep understanding of microelectronics and biochemistry. Avoiding post-CMOS fabrication and sample labeling, as well as building reusable biosensors can decrease the cost of the biosensors. Designing integrated sensors and sensing circuits with enhanced performance, taking advantage of sensing modalities that do not require sample labeling, and developing surface functionalization methods exploiting reversible chemical reactions are some strategies to achieve these goals.

## 7 Conclusion

We discussed surface-modified CMOS biosensors with different transduction mechanisms, such as electrochemical, optical, electrical, magnetic, mechanical, etc. CMOS electrochemical biosensing enables label-free detection, which reduces sample preparation steps, and allows for better integration of the sensor and sensing circuits. However, since the metal used in CMOS processes, aluminum, is not suitable for electrochemical sensing, post-CMOS processing steps are needed to fabricate working electrodes made of electrochemical-compatible material. Moreover, the development of CMOS electrochemical biosensors is facing other challenges such as Short Debye length, fabricating on-chip REs, poor specificity, and strong environmental influences that are yet to be addressed.

In CMOS optical biosensors, capture probes can be directly immobilized on the CMOS detector surface without the need for post-CMOS fabrication steps. Another advantage of optical biosensing is that it allows for both labeled and label-free detection. Between the two main optical detection methods, fluorescence-based and chemiluminescence-based, the former needs external light sources and objectives, while the latter does not (Hong L. et al., 2015; 2017; Kricka, 1995). Although developing on-chip filters is a challenging and costly task, fluorescence-based biosensors receive more attention, as fluorescence detection is the gold standard method for DNA detection. For more information on developing on-chip optical filters, the reader is encouraged to refer to (Dandin et al., 2007).

We also covered other sensing modalities for developing biosensors; i.e., electrical, magnetic, and mechanical. Magnetic biosensors’ advantage is their insensitivity to the matrix, which provides high sensitivity and specificity toward the target biomarker (Zhou et al., 2021); However, unlike electrochemical and optical biosensors, magnetic biosensors do not allow label-free detection, and it is more challenging to integrate them into a CMOS chip since they would need external permanent magnets or coils. To address the latter issue, efforts have been made to fabricate on-chip coils and LC oscillators. Furthermore, GMR SVs are compatible with CMOS technology and are promising candidates for developing fully integrated CMOS magnetic biosensors (Zhou et al., 2021). Mechanical biosensors did not emerge until the 1980s. Recently, CMOS microcantilever-based biosensors have been developed for molecular sensing applications. These biosensors offer high sensitivity, low limit of detection, fast-response, and portability (Zhao et al., 2021). Multimodal CMOS biosensors have multiple sensors with different transducing mechanisms on a single chip, making them suitable for monitoring cell activities (Tokuda et al., 2006; Al-Rawhani et al., 2020; Jung et al., 2021; Kumashi et al., 2021; Wang et al., 2022b).

Furthermore, we discussed materials and methods for modifying the surface of CMOS chips. These modifications are divided into two main categories; post-CMOS additive or subtractive fabrication processes, i.e., oxide etching and metal deposition, and biochemical processes for immobilizing the BRE, i.e., antibodies, on the chip’s surface. Finally, we discussed the future of the industry and the challenges facing the mass production of CMOS biosensors.
