# Supplementary material for: Surface-modified CMOS biosensors
Source: Front Bioeng Biotechnol. 2024 Nov 6;12:1441430. doi: 10.3389/fbioe.2024.1441430 (PMC11576298; doi:10.3389/fbioe.2024.1441430)
Supplement: Supplementary file 1 [file DataSheet2.PDF]

REFERENCES

Hassibi, A., Singh, R., Manickam, A., Sinha, R., Kuimelis, B., Bolouki, S., et al. (2017). A fully integrated CMOS fluorescence biochip for multiplex polymerase chain-reaction (PCR) processes. In *Digest of Technical Papers - IEEE International Solid-State Circuits Conference* (Institute of Electrical and Electronics Engineers Inc.), vol. 60, 68–69. doi:10.1109/ISSCC.2017.7870264

Ho, W. J., Chen, J. S., Ker, M. D., Wu, T. K., Wu, C. Y., Yang, Y. S., et al. (2007). Fabrication of a miniature CMOS-based optical biosensor. *Biosensors and Bioelectronics* 22, 3008–3013. doi:10.1016/j.bios.2006.12.031

Hofmann, A., Meister, M., Rolapp, A., Reich, P., Scholz, F., and Schäfer, E. (2020). Light-controlled photometer with optoelectronic CMOS biochip for quantitative PSA detection. *Proceedings - IEEE International Symposium on Circuits and Systems* 2020-Octob, 1–5. doi:10.1109/iscas45731.2020.9180796

Hofmann, A., Meister, M., Rolapp, A., Reich, P., Scholz, F., and Schafer, E. (2021). Light Absorption Measurement with a CMOS Biochip for Quantitative Immunoassay Based Point-of-Care Applications. *IEEE Transactions on Biomedical Circuits and Systems* 15, 369–379. doi:10.1109/TBCAS.2021.3083359

Hong, D., Joung, H. A., Lee, D. Y., Kim, S., and Kim, M. G. (2015a). Attomolar detection of cytokines using a chemiluminescence immunoassay based on an antibody-arrayed CMOS image sensor. *Sensors and Actuators, B: Chemical* 221, 1248–1255. doi:10.1016/j.snb.2015.07.042

Hong, L., Li, H., Yang, H., and Sengupta, K. (2017). Fully Integrated Fluorescence Biosensors On-Chip Employing Multi-Functional Nanoplasmonic Optical Structures in CMOS. *IEEE Journal of Solid-State Circuits* 52, 2388–2406. doi:10.1109/JSSC.2017.2712612

Hong, L., McManus, S., Yang, H., and Sengupta, K. (2015b). A fully integrated CMOS fluorescence biosensor with on-chip nanophotonic filter. In *IEEE Symposium on VLSI Circuits, Digest of Technical Papers* (Institute of Electrical and Electronics Engineers Inc.), vol. 2015-August, C206–C207. doi:10.1109/VLSIC.2015.7231260

Jang, B., Cao, P., Chevalier, A., Ellington, A., and Hassibi, A. (2009). A CMOS fluorescent-based biosensor microarray. In *Digest of Technical Papers - IEEE International Solid-State Circuits Conference*. doi:10.1109/ISSCC.2009.4977495

Joung, H. A., Hong, D. G., and Kim, M. G. (2012). A high sensitivity chemiluminescence-based CMOS image biosensor for the detection of human interleukin 5 (IL-5). In *Proceedings of IEEE Sensors*. doi:10.1109/ICSENS.2012.6411100

Liu, Q., Jimenez, M., Inda, M. E., Riaz, A., Zirtiloglu, T., Chandrakasan, A. P., et al. (2022). A Threshold-Based Bioluminescence Detector With a CMOS-Integrated Photodiode Array in 65 nm for a Multi-Diagnostic Ingestible Capsule. *IEEE Journal of Solid-State Circuits* , 1–14doi:10.1109/JSSC.2022.3197465

Manickam, A., Johnson, K. A., Singh, R., Wood, N., Ku, E., Cuppoletti, A., et al. (2021). Multiplex PCR CMOS Biochip for Detection of Upper Respiratory Pathogens including SARS-CoV-2. *IEEE Symposium on VLSI Circuits, Digest of Technical Papers* 2021-June, 1–2. doi:10.23919/VLSICircuits52068.2021.9492353

Manickam, A., Singh, R., Mcdermott, M. W., Wood, N., Bolouki, S., Naraghi-Arani, P., et al. (2017). A fully integrated CMOS fluorescence biochip for DNA and RNA testing. *IEEE Journal of Solid-State Circuits* 52, 2857–2870. doi:10.1109/JSSC.2017.2754363

Sandeau, L., Vuillaume, C., Contié, S., Grinenval, E., Belloni, F., Rigneault, H., et al. (2015). Large area CMOS bio-pixel array for compact high sensitive multiplex biosensing. *Lab on a Chip* 15, 877–881. doi:10.1039/c4lc01025f

Stadler, V., Beyer, M., König, K., Nesterov, A., Torralba, G., Lindenstruth, V., et al. (2007). Multifunctional CMOS microchip coatings for protein and peptide arrays. *Journal of Proteome Research* 6, 3197–3202. doi:10.1021/pr0701310

Zhu, C., Hong, L., Yang, H., and Sengupta, K. (2022). A Packaged Multiplexed Fluorescent Bio-molecular Sensor Array and Ultra-Low-Power Wireless Interface in CMOS for Ingestible Electronic Applications. *IEEE Sensors Journal* 22, 1–1. doi:10.1109/jsen.2022.3216455

Table 2. Performance summary of optical biosensors

Transducer<br>Sensing param.	Surface material/ modification	Target<br>Biological sample<br>BRE	LOD<br>Sensitivity<br>Range	Ref.
PD<br>Fluorescence	SiO_2	DNA<br>SARS–CoV–2, FluA, FluB, RSV<br>probe DNA	–<br>–<br>–	(Manickam et al., 2021)
PD<br>Fluorescence	SiO_2	–<br>FluA, FluB, RSV, HPIV, AdvC2, AdvE, polio<br>–	5×10^{10} ph/(cm^2.sec)*<br>–<br>10 fA – 10 nA	(Hassibi et al., 2017)
PD<br>Fluorescence	SiO_2	–<br>FluA, FluB, RSV, HPIV, AdvC2, AdvE, polio<br>–	5×10^{10} ph/(cm^2.sec)*<br>–<br>10 fA – 10 nA	(Manickam et al., 2017)
PD<br>Fluorescence	SiO_2	DNA sequence<br>–<br>–	10^3 ph/(cm^2.sec)*<br>–<br>10 fA – 10 nA	(Jang et al., 2009)
PD<br>Fluorescence	BEOL layer	streptavidin	804 fW<br>47 zeptomoles of Qdots	(Hong et al., 2015b)
PD<br>Fluorescence	BEOL layer	streptavidin	804 fW<br>48 zeptomoles of Qdots	(Hong et al., 2017)
CMOS Image Sensor<br>Fluorescence	PEGMA film	–<br>anti–rabbit IgG (H+L), goat anti–mouse IgG (H+L)<br>anti–FLAG	–<br>–	(Stadler et al., 2007)
PD<br>fluorescence	indigestible capsule	GI tract monitoring<br>porcine belly tissue<br>–	zeptomole<br>pM<br>–	(Zhu et al., 2022)
CMOS image sensor<br>Chemiluminescence	GOPTS	TNLα, IL8, IFN$_7$	3 pg/ml<br>–	(Sandeau et al., 2015)
CMOS image sensor<br>Chemiluminescence	sensor surface	interlukin–5	0.1 pg/ml	(Joung et al., 2012)
CMOS image sensor<br>Chemiluminescence	Si_3N_4	interlukin–2, 4, 5, 6	0.074 fg/ml	(Hong et al., 2015a)
PD<br>Luminescence	PD surface	H_2O_2, glucose	0.05 mM H_2O_2, 0.5 mM glucose<br>0.05 – 20 mM H_2O_2, 0.5 – 20 mM glucose	(Ho et al., 2007)
PD<br>Light absorption	bio–compatible chip–on–board package	Antigen<br>PSA<br>capture antibody	0.5 ng/ml<br>–<br>–	(Hofmann et al., 2020, 2021)

PD	indigestible	bacteria	59 fA	
Bioluminescence	capsule	tetrathionate	–	(Liu et al., 2022)
		–	–	
