# Supplementary material for: Surface-modified CMOS biosensors
Source: Front Bioeng Biotechnol. 2024 Nov 6;12:1441430. doi: 10.3389/fbioe.2024.1441430 (PMC11576298; doi:10.3389/fbioe.2024.1441430)
Supplement: Supplementary file 2 [file DataSheet3.PDF]

REFERENCES

Al-Rawhani, M. A., Mitra, S., Barrett, M. P., Cochran, S., Cumming, D. R., Hu, C., et al. (2020). Multimodal Integrated Sensor Platform for Rapid Biomarker Detection. *IEEE Transactions on Biomedical Engineering* 67, 614–623. doi:10.1109/TBME.2019.2919192

Chen, C. H., Hwang, R. Z., Huang, L. S., Lin, S. M., Chen, H. C., Yang, Y. C., et al. (2009). A wireless Bio-MEMS sensor for C-reactive protein detection based on nanomechanics. *IEEE Transactions on Biomedical Engineering* 56, 462–470. doi:10.1109/TBME.2008.2003262

Costa, T., Cardoso, F. A., Germano, J., Freitas, P. P., and Piedade, M. S. (2017). A CMOS Front-End with Integrated Magnetoresistive Sensors for Biomolecular Recognition Detection Applications. *IEEE Transactions on Biomedical Circuits and Systems* 11, 988–1000. doi:10.1109/TBCAS.2017.2743685

Fuller, C. W., Padayatti, P. S., Abderrahim, H., Adamiak, L., Alagar, N., Ananthapadmanabhan, N., et al. (2022). Molecular electronics sensors on a scalable semiconductor chip: A platform for single-molecule measurement of binding kinetics and enzyme activity. *PNAS* 119. doi:10.1073/pnas.2112812119/-/DCSupplemental

Gambini, S., Skucha, K., Liu, P. P., Kim, J., and Krigel, R. (2013). A 10 kPixel CMOS hall sensor array with baseline suppression and parallel readout for immunoassays. *IEEE Journal of Solid-State Circuits* 48, 302–317. doi:10.1109/JSSC.2012.2224531

Hall, D. A., Gaster, R. S., Makinwa, K. A., Wang, S. X., and Murmann, B. (2013). A 256 pixel magnetoresistive biosensor microarray in 0.18 μm CMOS. *IEEE Journal of Solid-State Circuits* 48, 1290–1301. doi:10.1109/JSSC.2013.2245058

Han, S. J., Xu, L., Yu, H., Wilson, R. J., White, R. L., Pourmand, N., et al. (2006). CMOS integrated DNA microarray based on GMR sensors. In *Technical Digest - International Electron Devices Meeting, IEDM*. doi:10.1109/IEDM.2006.346887

Hu, K., Arcadia, C. E., and Rosenstein, J. K. (2021). A large-scale multimodal CMOS biosensor array with 131,072 pixels and code-division multiplexed readout. *IEEE Solid-State Circuits Letters* 4, 48–51. doi:10.1109/LSSC.2021.3056515

Huang, C. W., Hsueh, H. T., Huang, Y. J., Liao, H. H., Tsai, H. H., Juang, Y. Z., et al. (2013). A fully integrated wireless CMOS microcantilever lab chip for detection of DNA from Hepatitis B virus (HBV). *Sensors and Actuators, B: Chemical* 181, 867–873. doi:10.1016/j.snb.2013.02.061

Jung, D., Junek, G. V., Park, J. S., Kumashi, S. R., Wang, A., Li, S., et al. (2021). A CMOS 21 952-Pixel Multi-Modal Cell-Based Biosensor with Four-Point Impedance Sensing for Holistic Cellular Characterization. *IEEE Journal of Solid-State Circuits* 56, 2438–2451. doi:10.1109/JSSC.2021.3085571

Kumashi, S., Jung, D., Park, J., Tejedor-Sanz, S., Grijalva, S., Wang, A., et al. (2021). A CMOS Multi-Modal Electrochemical and Impedance Cellular Sensing Array for Massively Paralleled Exoelectrogen Screening. *IEEE Transactions on Biomedical Circuits and Systems* 15, 221–234. doi:10.1109/TBCAS.2021.3068710

Lai, S., Caboni, A., Loi, D., and Barbaro, M. (2012). A CMOS biocompatible charge detector for biosensing applications. *IEEE Transactions on Electron Devices* 59, 2512–2519. doi:10.1109/TED.2012.2202233

Li, J., Xue, M., Lu, Z., Zhang, Z., Feng, C., and Chan, M. (2003). A high-density conduction-based micro-DNA identification array fabricated with a CMOS compatible process. *IEEE Transactions on Electron Devices* 50, 2165–2170. doi:10.1109/TED.2003.816545

Li, Y., Vancura, C., Kirstein, K. U., Lichtenberg, J., and Hierlemann, A. (2008). Monolithic resonant-cantilever-based CMOS microsystem for biochemical sensing. *IEEE Transactions on Circuits and Systems I: Regular Papers* 55, 2551–2560. doi:10.1109/TCSI.2008.922027

Maruyama, Y., Terao, S., and Sawada, K. (2009). Label free CMOS DNA image sensor based on the charge transfer technique. *Biosensors and Bioelectronics* 24, 3108–3112. doi:10.1016/j.bios.2009.03.031

Tokuda, T., Yamamoto, A., Kagawa, K., Nunoshita, M., and Ohta, J. (2006). A CMOS image sensor with optical and potential dual imaging function for on-chip bioscientific applications. *Sensors and Actuators, A: Physical* 125, 273–280. doi:10.1016/j.sna.2005.08.023

Wang, A. Y., Sheng, Y., Li, W., Jung, D., Junek, G., Park, J., et al. (2022). A CMOS Cellular Interface Array for Digital Physiology Featuring High-Density Multi-Modal Pixels and Reconfigurable Sampling Rate. *Digest of Technical Papers - IEEE International Solid-State Circuits Conference* 2022-February, 202–204. doi:10.1109/ISSCC42614.2022.9731629

Wang, H., Mahdavi, A., Park, J., Chi, T., Butts, J., Hookway, T. A., et al. (2014). Cell culture and cell based sensor on CMOS. In *IEEE 2014 Biomedical Circuits and Systems Conference, BioCAS 2014 - Proceedings* (Institute of Electrical and Electronics Engineers Inc.), 468–471. doi:10.1109/BioCAS.2014.6981764

Wang, L., Fu, D., Liu, X., Zhao, J., Zhao, J., Yuan, Q., et al. (2021). Highly sensitive biosensor based on a microcantilever and alternating current electrothermal technology. *Journal of Micromechanics and Microengineering* 31. doi:10.1088/1361-6439/abcae6

Zhao, J., Wang, L., Fu, D., Zhao, D., Wang, Y., Yuan, Q., et al. (2021). Gold nanoparticles amplified microcantilever biosensor for detecting protein biomarkers with high sensitivity. *Sensors and Actuators, A: Physical* 321. doi:10.1016/j.sna.2021.112563

Table 3. Performance summary of other biosensors

Transducer / Sensing mechanism	Surface material/ modification	Target / Biological sample / BRE	LOD / Sensitivity / Range	Ref.
PMOS-NMOS FG–FET Electrical	Al_2O_3	DNA<br>–<br>probe DNA	10^{-14} C<br>–<br>10^{-10}–10^{-14} C	(Lai et al., 2012)
FET Electrical	Si_3Ni_4	DNA<br>22 bases DNA<br>probe DNA	2.7×10^7 molecules/cm^2<br>–<br>–	(Maruyama et al., 2009)
CMOS DNA array Electrical	SiO_2	DNA<br>–<br>probe DNA	1 pM<br>–<br>1 pM – 300 nM	(Li et al., 2003)
Molecular electronics Electrical	Wire bridge	Biomolecule<br>–<br>probe biomolecule	–<br>single molecule<br>–	(Fuller et al., 2022)
LC resonator Magnetic	Gelatin/Si_3Ni_4	Cell<br>Mouse/H7 ESC<br>probe molecules	sub–ppm<br>–<br>–	(Wang et al., 2014)
GMR sensor Magnetic	SiO_2	Protein<br>Streptavidin<br>probe DNA	0.1 Oe<br>single nanoparticle<br>–	(Han et al., 2006)
GMR sensor Magnetic	AlN/SV	Biomolecule<br>–<br>–	–<br>MR_{Ratio} = 5.37%<br>–	(Costa et al., 2017)
GMR SV Magnetic	Oxide	Protein<br>SLPI<br>Capture antibody	10 fM<br>–<br>3.5 OOM	(Hall et al., 2013)
Hall sensor Magnetic	Top metal	Protein<br>HSA<br>Capture antibody	200 pg/ml<br>200 pg/ml<br>–	(Gambini et al., 2013)
μcantilever Mechanical	–	Protein<br>PSA<br>antiPSA	30 pg<br>–<br>–	(Li et al., 2008)
μcantilever Mechanical	Au/Cr/Si_3Ni_4	Protein<br>anti–CRP<br>–	1 μg/ml<br>–<br>1 μg/ml – 500 μg/ml	(Chen et al., 2009)
μcantilever Mechanical	Au	Virus<br>HBV<br>probe DNA	1 pM<br>1 pM - 10 nM	(Huang et al., 2013)
μcantilever Mechanical	SiO_2	Protein<br>AFP<br>Capture antibody	21 pg/ml<br>–<br>0–70 ng/ml	(Zhao et al., 2021)
μcantilever Mechanical	Au	Bacteria<br>Vibrio parahaemolyticus<br>capture antibody	5×10^5 CFU/ml<br>–<br>$5\times 10^5 - 10^7$ CFU/ml	(Wang et al., 2021)
MOSFET, PD Multimodal	Color filter<br>DNA spot	DNA, Neural cell imaging<br>–<br>–	–<br>1.6 mV<br>–	(Tokuda et al., 2006)
TiN ISFET, PD Multimodal	exposed TiN	Bacteria imaging<br>–<br>–	–<br>0.7 aF, 27.7 mV/pH<br>–	(Hu et al., 2021)

Electrode, PD<br>Multimodal	Au/Ti/Al	Cellular charac.<br>–<br>–	–<br>Single–cell<br>–	(Jung et al., 2021)
Electrode<br><br>Multimodal	Au/Ti/Al<br><br>PEDOT:PSS/<br>AgCl/Ag/Au/Ti	Exoelectrogen<br>Screening<br>Bacteria, HEK–293<br>–	–<br><br>0.204 pA<br>–	(Kumashi et al., 2021)
Electrode, PD<br>Multimodal	Pt/Ti/Al	Biosensing/actuating<br>SNaPs,C2C12<br>–	–<br>Single–cell<br>–	(Wang et al., 2022)
PD, SPAD, ISFET<br>Multimodal	SiO_2<br>$Au/SiO_2/$<br><br>polymide/<br>Si_3Ni_4/SiO_2	metabolite<br>glucose,cholesterol,<br><br>urea,urate<br>–	0.45 nW/cm^2<br>33 mV/pH,7.3 Mcps/RIU<br>pH 5 – 9,<br>physiological range	(Al-Rawhani et al., 2020)
