# Supplementary material for: Surface-modified CMOS biosensors
Source: Front Bioeng Biotechnol. 2024 Nov 6;12:1441430. doi: 10.3389/fbioe.2024.1441430 (PMC11576298; doi:10.3389/fbioe.2024.1441430)
Supplement: Supplementary file 3 [file DataSheet1.PDF]

REFERENCES

Andrianova, M. S., Kuznetsov, E. V., Grudtsov, V. P., and Kuznetsov, A. E. (2018). CMOS-compatible biosensor for L-carnitine detection. *Biosensors and Bioelectronics* 119, 48–54. doi:10.1016/j.bios.2018.07.044

Chang, C. F. and Lu, M. S. (2020). CMOS Ion Sensitive Field Effect Transistors for Highly Sensitive Detection of DNA Hybridization. *IEEE Sensors Journal* 20, 8930–8937. doi:10.1109/JSEN.2020.2986461

Chua, J. H., Chee, R. E., Agarwal, A., She, M. W., and Zhang, G. J. (2009). Label-free electrical detection of cardiac biomarker with complementary metal-oxide semiconductor-compatible silicon nanowire sensor arrays. *Analytical Chemistry* 81, 6266–6271. doi:10.1021/ac901157x

Doi, H., Horio, T., Choi, Y. J., Takahashi, K., Noda, T., and Sawada, K. (2022). CMOS-based redox-type label-free ATP image sensor for in vitro sensitive imaging of extracellular ATP. *Sensors* 22. doi:10.3390/s22010075

Dudina, A., Frey, U., and Hierlemann, A. (2019). Carbon-nanotube-based monolithic CMOS platform for electrochemical detection of neurotransmitter glutamate. *Sensors (Switzerland)* 19. doi:10.3390/s19143080

Gao, A., Lu, N., Wang, Y., and Li, T. (2016). Robust ultrasensitive tunneling-FET biosensor for point-of-care diagnostics. *Scientific Reports* 6. doi:10.1038/srep22554

Hsu, C. L., Sun, A., Zhao, Y., Aronoff-Spencer, E., and Hall, D. A. (2018). A 16×20 electrochemical CMOS biosensor array with in-pixel averaging using polar modulation. In *2018 IEEE Custom Integrated Circuits Conference, CICC 2018* (Institute of Electrical and Electronics Engineers Inc.), 1–4. doi:10.1109/CICC.2018.8357044

Huang, C. W., Huang, Y. J., Yen, P. W., Tsai, H. H., Liao, H. H., Juang, Y. Z., et al. (2013). A CMOS wireless biomolecular sensing system-on-chip based on polysilicon nanowire technology. *Lab on a Chip* 13, 4451–4459. doi:10.1039/c3lc50798j

Krivitsky, V., Zverzhinetsky, M., Krivitsky, A., Hsiung, L. C., Naddaka, V., Gabriel, I., et al. (2019). Cellular Metabolomics by a Universal Redox-Reactive Nanosensors Array: From the Cell Level to Tumor-on-a-Chip Analysis. *Nano Letters* 19, 2478–2488. doi:10.1021/acs.nanolett.9b00052

Kuo, P. Y., Chen, Y. Y., Lai, W. H., and Chang, C. H. (2021). An Extended-Gate Field-Effect Transistor Applied to Resistive Divider Integrated with the Readout Circuit Using 180nm CMOS Process for Uric Acid Detection. *IEEE Sensors Journal* 21, 20229–20238. doi:10.1109/JSEN.2021.3093642

Kuo, Y. H., Chen, Y. S., Huang, P. C., and Lee, G. B. (2020). A CMOS-Based Capacitive Biosensor for Detection of a Breast Cancer MicroRNA Biomarker. *IEEE Open Journal of Nanotechnology* 1, 157–162. doi:10.1109/OJNANO.2020.3035349

Lai, W. A., Lin, C. H., Yang, Y. S., and Lu, M. S. (2012). Ultrasensitive detection of avian influenza virus by using CMOS impedimetric sensor arrays. In *Proceedings of the IEEE International Conference on Micro Electro Mechanical Systems (MEMS)*. 894–897. doi:10.1109/MEMSYS.2012.6170329

Lee, B. Y., Seo, S. M., Lee, D. J., Lee, M., Lee, J., Cheon, J. H., et al. (2010). Biosensor system-on-a-chip including CMOS-based signal processing circuits and 64 carbon nanotube-based sensors for the detection of a neurotransmitter. *Lab on a Chip* 10, 894–898. doi:10.1039/b916975j

Lee, C., Chen, Y. W., and Lu, M. S. (2021). CMOS Biosensors for the Detection of DNA Hybridization in High Ionic-Strength Solutions. *IEEE Sensors Journal* 21, 4135–4142. doi:10.1109/JSEN.2020.3031321

Lee, K. H., Choi, S., Lee, J. O., Yoon, J. B., and Cho, G. H. (2012). CMOS capacitive biosensor with enhanced sensitivity for label-free DNA detection. In *Digest of Technical Papers - IEEE International Solid-State Circuits Conference*. vol. 55, 120–121. doi:10.1109/ISSCC.2012.6176945

Li, D.-C., Yang, P.-H., and Lu, M. S.-C. (2010). CMOS Open-Gate Ion-Sensitive Field-Effect Transistors for Ultrasensitive Dopamine Detection _ Enhanced Reader. *IEEE transactions on electron devices* 57, 2761–2767

Lin, C. H., Hung, C. H., Hsiao, C. Y., Lin, H. C., Ko, F. H., and Yang, Y. S. (2009). Poly-silicon nanowire field-effect transistor for ultrasensitive and label-free detection of pathogenic avian influenza DNA. *Biosensors and Bioelectronics* 24, 3019–3024. doi:10.1016/j.bios.2009.03.014

Lin, C. H., Lee, Y. C., Yang, C. M., and Lu, M. S. (2022). Detection of DNA Hybridization Beyond the Debye Screening Length by CMOS Capacitive Sensors. *IEEE Electron Device Letters* 43, 1319–1322. doi:10.1109/LED.2022.3182784

Lin, H.-H., Wang, I.-S., Yen, P.-W., Cheng, H., Tsai, H.-H., Liao, H.-H., et al. (2014). A CMOS Based Polysilicon Nanowire Biosensor Platform for Different Biological Targets. *Procedia Engineering* 87, 340–343. doi:10.1016/j.proeng.2014.11.752

Manickam, A., You, K.-D., Wood, N., Pei, L., Liu, Y., Singh, R., et al. (2019a). A CMOS Biosensor Array with 1024 3-Electrode }Voltammetry Pixels and 93dB Dynamic Range. *IEEE International Solid-State Circuits Conference* , 192–194

Manickam, A., You, K. D., Wood, N., Pei, L., Liu, Y., Singh, R., et al. (2019b). A CMOS Electrochemical Biochip with 32 × 32 Three-Electrode Voltammetry Pixels. *IEEE Journal of Solid-State Circuits* 54, 2980–2990. doi:10.1109/JSSC.2019.2941020

Mirsian, S., Khodadadian, A., Hedayati, M., Manzour-ol Ajdad, A., Kalantarinejad, R., and Heitzinger, C. (2019). A new method for selective functionalization of silicon nanowire sensors and Bayesian inversion for its parameters. *Biosensors and Bioelectronics* 142. doi:10.1016/j.bios.2019.111527

Moser, N., Rodriguez-Manzano, J., Lande, T. S., and Georgiou, P. (2018). A scalable ISFET sensing and memory array with sensor auto-calibration for on-chip real-time DNA detection. *IEEE Transactions on Biomedical Circuits and Systems* 12, 390–401. doi:10.1109/TBCAS.2017.2789161

Nasri, B., Wu, T., Alharbi, A., Gupta, M., Ranjitkumar, R., Sebastian, S., et al. (2017). Heterogeneous integrated CMOS-graphene sensor array for dopamine detection. In *Digest of Technical Papers - IEEE International Solid-State Circuits Conference* (Institute of Electrical and Electronics Engineers Inc.), vol. 60, 268–269. doi:10.1109/ISSCC.2017.7870364

Nikkhoo, N., Cumby, N., Gulak, P. G., and Maxwell, K. L. (2016). Rapid bacterial detection via an all-electronic CMOS biosensor. *PLoS ONE* 11. doi:10.1371/journal.pone.0162438

Pei-Wen, Y., Che-Wei, H., Yu-Jie, H., Min-Cheng, C., Hsin-Hao, L., Shey-Shi, L., et al. (2014). A device design of an integrated CMOS poly-silicon biosensor-on-chip to enhance performance of biomolecular analytes in serum samples. *Biosensors and Bioelectronics* 61, 112–118. doi:10.1016/j.bios.2014.05.010

Saengdee, P., Thanapitak, S., Ongwattanakul, S., Srisuwan, A., Pankiew, A., Thornyanadacha, N., et al. (2021). A silicon nitride ion sensitive field effect transistor-based immunosensor for determination of urinary albumin. *Electrochemical Science Advances* doi:10.1002/elsa.202100078

Senevirathna, B. P., Lu, S., Dandin, M. P., Basile, J., Smela, E., and Abshire, P. A. (2018). Real-Time Measurements of Cell Proliferation Using a Lab-on-CMOS Capacitance Sensor Array. *IEEE Transactions on Biomedical Circuits and Systems* 12, 510–520. doi:10.1109/TBCAS.2018.2821060

Sessi, V., Ibarlucea, B., Seichepine, F., Klinghammer, S., Ibrahim, I., Heinzig, A., et al. (2022). Multisite Dopamine Sensing With Femtomolar Resolution Using a CMOS Enabled Aptasensor Chip. *Frontiers in Neuroscience* 16. doi:10.3389/fnins.2022.875656

Shariati, M. (2018). The field effect transistor DNA biosensor based on ITO nanowires in label-free hepatitis B virus detecting compatible with CMOS technology. *Biosensors and Bioelectronics* 105, 58–64. doi:10.1016/j.bios.2018.01.022

Sheibani, S., Capua, L., Kamaei, S., Akbari, S. S. A., Zhang, J., Guerin, H., et al. (2021). Extended gate field-effect-transistor for sensing cortisol stress hormone. *Communications Materials* 2. doi:10.1038/s43246-020-00114-x

Singh, N. K., Thungon, P. D., Estrela, P., and Goswami, P. (2019). Development of an aptamer-based field effect transistor biosensor for quantitative detection of Plasmodium falciparum glutamate dehydrogenase in serum samples. *Biosensors and Bioelectronics* 123, 30–35. doi:10.1016/j.bios.2018.09.085

Stern, E., Klemic, J. F., Routenberg, D. A., Wyrembak, P. N., Turner-Evans, D. B., Hamilton, A. D., et al. (2007). Label-free immunodetection with CMOS-compatible semiconducting nanowires. *Nature* 445, 519–522. doi:10.1038/nature05498

Tabrizi, H. O., Forouhi, S., Ghafar-Zadeh, M., Magierowski, S., and Ghafar-Zadeh, E. (2021). CMOS Capacitive DNA Nano-Mass Measurement for DNA Storage Application. In *Canadian Conference on Electrical and Computer Engineering* (Institute of Electrical and Electronics Engineers Inc.), vol. 2021-September. doi:10.1109/CCECE53047.2021.9569194

Yao, L., Hajj-Hassan, M., Ghafar-Zadeh, E., Shabani, A., Chodavarapu, V., and Zourob, M. (2008). CMOS capactive sensor system for bacteria detection using phage organisms. In *Canadian Conference on Electrical and Computer Engineering*. 877–880. doi:10.1109/CCECE.2008.4564661

Yong, S. K., Shen, S. K., Chiang, C. W., Weng, Y. Y., Lu, M. P., and Yang, Y. S. (2021). Silicon nanowire field-effect transistor as label-free detection of hepatitis b virus proteins with opposite net charges. *Biosensors* 11. doi:10.3390/bios11110442

Zhang, J., Rupakula, M., Bellando, F., Garcia Cordero, E., Longo, J., Wildhaber, F., et al. (2019). Sweat Biomarker Sensor Incorporating Picowatt, Three-Dimensional Extended Metal Gate Ion Sensitive Field Effect Transistors. *ACS Sensors* 4, 2039–2047. doi:10.1021/acssensors.9b00597

Table 1. Performance summary of electrochemical biosensors

Transducer / Sensing param.	Surface material/ modification	Target / Biological sample / BRE	LOD / Sensitivity / Range	Ref.
3–electrode<br>Voltametry	amorphous<br>carbon	DNA<br>–<br>DNA hairpin probe	70 nM MB<br>4 pA/μM<br>–	(Manickam et al., 2019a,b)
3–electrode<br>Voltametry	graphene<br>sheet	Neurotransmitter<br>dopamine<br>–	–<br>8–14 nA/μM<br>–	(Nasri et al., 2017)
Au electrode<br>Potentiometric	enzyme<br>membrane on<br>Au electrodes	Neurotransmitter<br>ATP<br>GK, LGOx, HRP	28 μM<br>77± 3.8 mV/dec	(Doi et al., 2022)
swCNT–based sensor<br>conductance	swCNT<br>adsorbed on<br>SiO_2 and Al	Neurotransmitter<br>glutamate<br>GOx	–<br>–<br>–	(Lee et al., 2010)
comb–shaped IDE<br>Capacitance	Au IDE	Cancer biomarker<br>microRNA–195<br>probe DNA	0.617 fM<br>665 Hz/fM<br>10 fM – 10 pM	(Kuo et al., 2020)
IDE<br>Capacitance	top metal<br>layer/<br>passivation	cell monitoring<br>human ovarian cancer cell<br>–	14.4 aF<br>100 aF/cell<br>–	(Senevirathna et al., 2018)
Au IDE<br>Capacitance	Au IDE	Virus<br>H5N1<br>probe DNA	0.1 nM<br>1.69 pulses/pF<br>0.1 nM – 1 μM	(Lee et al., 2012)
IDE<br>Capacitance	Thinned<br>inter–metal<br>SiO_2	Virus<br>HBV<br>probe DNA	1 fM<br>10.8 fF/log[DNA]<br>1–100 fM	(Lin et al., 2022)
Metal IDE<br>Capacitance	Top metal<br>layer	Bacteria<br>Escherichia coli<br>Bacteriophage	–<br>29.3 mV/fF<br>–	(Yao et al., 2008)
Metal IDE<br>Capacitance	Al_2O_3<br>passivation<br>layer	DNA storage<br>dried DNA nmass<br>–	–<br>18.5 aF/ng<br>–	(Tabrizi et al., 2021)
ISFET<br>Capacitance	inter–metal<br>oxide	Virus<br>HBV<br>probe DNA	1 pM<br>–<br>1 pM – 10 nM	(Lee et al., 2021)
Au electrodes<br>Capacitance	Au/Ni	Virus<br>Zika<br>probe DNA	–<br>–<br>5 kHz – 1 MHz	(Hsu et al., 2018)
IDμE FET<br>Capacitance	SiO_2	Virus<br>AIV H5<br>probe DNA	1 fM<br>–15.8%ΔC<br>1 fM – 10 fM	(Lai et al., 2012)
aptaFET<br>Δ V_{GS}	AuμE	Protein<br>PfGDH<br>ssDNA aptamer	48.6 pM<br>–<br>100 fM – 10 nM	(Singh et al., 2019)
Si_3Ni_4–ISFET<br>Δ V_{GS}	Si_3Ni_4	Antibody/Antigen<br>HSA<br>Antibody	–<br>5 μg/ml<br>5 – 500 μg/ml	(Saengdee et al., 2021)
ISFET<br>Δfrequency, Δ V_{th}	SiO_2	Virus<br>HBV<br>probe DNA	–<br>32 mv/pH<br>1 – 10 fM	(Chang and Lu, 2020)

3D–EMG–ISFET<br>Δ V_{th}	Al_2O_3, Si_3Ni_4	Sweat biomarker<br>$H^+/Na^+/K^+/Ca^{2+}$<br>$(Na^+/K^+/Ca^{2+})$ sensitive membrane	1 mM<br>58 mV/pH, –57/–48/–26 mV/dec<br>–	(Zhang et al., 2019)
ISFET<br>ΔV_{GS}	Si_3N_4/SiO_2	DNA<br>lambda phage DNA<br>–	–<br>3.2 μs/pH<br>–	(Moser et al., 2018)
ISFET<br>ΔV_{th}	ultrathin Ta_2O_5	Enzyme<br>L–carnitine<br>carnitine acetyltransferase	0.2 μM<br>18.0 ± 1.7 mV/μ M<br>0.2 – 50 μM	(Andrianova et al., 2018)
ISFET<br>ΔV_{th}	Potassium–sensitive membrane	Bacteria<br>Escherichia coli<br>valinomycin–PVC	10^{-6} M<br>3 × 10^8 bacteria/mL<br>10^{-5} – 10^{-2}	(Nikkhoo et al., 2016)
ISFET<br>Current	Si_3Ni_4/SiO_2	Neurotransmitter<br>dopamine<br>CPBA	1 fM<br>–<br>1 fM – 25 fM	(Li et al., 2010)
EG–FET<br>ΔV_{th}	Graphene	Hormone<br>cortisol<br>aptamer	0.2 nM<br>14 mV/dec<br>1 nM – 10 μ M	(Sheibani et al., 2021)
EG–FET<br>ΔV_{th}	RuO_2	Enzyme<br>Uric acid<br>Uricase	0.082 mg/dL<br>12.69 mV/(mg/dL)<br>–	(Kuo et al., 2021)
poly–Si NW–FET<br>Current	poly–Si	Virus<br>H5 AI<br>probe DNA	100 fM<br>–<br>100 fM – 100 PM	(Lin et al., 2009)
ITO NW–FET<br>Current	Au desorated ITO NW	Virus<br>HBV<br>probe DNA	1 fM<br>–<br>1 fM – 10 μM	(Shariati, 2018)
Si NW–FET<br>Current	Si NW	Protein<br>PSA<br>PSA antibody	90 pg/mL<br>–<br>1 – 100 ng/mL	(Mirsian et al., 2019)
poly–Si NW–FET<br>ΔV_{th}	poly–Si	Virus<br>HBsAg/HBx<br>HBsAb	3.92 fM/ 5.61 fM<br>–<br>3.92 fM – 0.39 pM/ 5.61 fM – 0.56 pM	(Yong et al., 2021)
Bottom–gate poly–Si NW–FET<br>Voltage	poly–Si	Protein<br>cTnI<br>anti–cTnI	0.32 pM<br>–<br>0.32 pM – 0.32 nM	(Pei-Wen et al., 2014)
Si NW–FET<br>Current	Si	Protein<br>Streptavidin<br>Biotin	10 fM<br>–<br>10 fM – 1 nM	(Stern et al., 2007)
Si NW–TFET<br>Current	Si	Cancer biomarker<br>CYFRA21–1<br>anti–CYFRA21–1	12.5 aM<br>–<br>0.5 fg/ml – 10 ng/ml	(Gao et al., 2016)
poly–Si NW–FET<br>Current	SiO_2	Protein<br>HBV/cTNI<br>probe DNA	10 fM/3.2 pM<br>–<br>3.2 pM – 320 pM	(Huang et al., 2013)
poly–Si NW–FET<br>Voltage	poly–Si	Protein<br>NT–proBNP, LDL,Hb,HbA1C<br>Antibody	32 pM, 1 μg/ml,100 ng/ml, 4 nM<br>–<br>32 pM – 32 nM,1 μg/ml – 1 mg/ml,100 ng/ml – 100 μg/ml, 4 nM – 400 nM	(Lin et al., 2014)

Si NW–FET<br>Conductance	Si	Protein<br>cTnT<br>anti–cTnI	1 fg/mL<br>–<br>1 fg/mL – 1 ng/mL	(Chua et al., 2009)
Si NW–FET<br>Current	Si	Metabolite<br>choline,glucose,pyruvat<br>DHA/AQ	100 nM<br>100 nM to H_2O_2<br>physiological range	(Krivitsky et al., 2019)
Si NW SJ–FET<br>Voltage	HfO_2	Neurotransmitter<br>dopamine<br>DNA aptamers	fM range<br>1 V/fM<br>–	(Sessi et al., 2022)
CNT–FET	CNT electrodes	Neurotransmitter<br>glutamate<br>GIOx	10 μM<br>–<br>–	(Dudina et al., 2019)
